# Comparative chloroplast genomics, phylogenetic relationships and molecular markers development of *Aglaonema commutatum* and seven green cultivars of *Aglaonema*

**DOI:** 10.1038/s41598-024-62586-y

**Published:** 2024-05-23

**Authors:** Dong-Mei Li, Yan-Gu Pan, Xiao-Ye Wu, Shui-Ping Zou, Lan Wang, Gen-Fa Zhu

**Affiliations:** 1 Guangdong Key Lab of Ornamental Plant Germplasm Innovation and Utilization, Environmental Horticulture Research Institute, Guangdong Academy of Agricultural Sciences, Guangzhou, China; 2 Research Institute of Living Environment, Guangdong Bailin Ecology and Technology Co., LTD, Dongguan, China

**Keywords:** *Aglaonema*, Chloroplast genome, Comparative genomics, Phylogenetic relationships, Molecular markers, Computational biology and bioinformatics, Molecular biology, Plant sciences

## Abstract

*Aglaonema commutatum* is a famous species in the *Aglaonema* genus, which has important ornamental and economic value. However, its chloroplast genome information and phylogenetic relationships among popular green cultivars of *Aglaonema* in southern China have not been reported. Herein, chloroplast genomes of one variety of *A. commutatum* and seven green cultivars of *Aglaonema*, namely, *A. commutatum* ‘San Remo’, ‘Kai Sa’, ‘Pattaya Beauty’, ‘Sapphire’, ‘Silver Queen’, ‘Snow White’, ‘White Gem’, and ‘White Horse Prince’, were sequenced and assembled for comparative analysis and phylogeny. These eight genomes possessed a typical quadripartite structure that consisted of a LSC region (90,799–91,486 bp), an SSC region (20,508–21,137 bp) and a pair of IR regions (26,661–26,750 bp). Each genome contained 112 different genes, comprising 79 protein-coding genes, 29 tRNA genes and 4 rRNA genes. The gene orders, GC contents, codon usage frequency, and IR/SC boundaries were highly conserved among these eight genomes. Long repeats, SSRs, SNPs and indels were analyzed among these eight genomes. Comparative analysis of 15 *Aglaonema* chloroplast genomes identified 7 highly variable regions, including *trnH-GUG-exon1-psbA*, *trnS-GCU-trnG-UCC-exon1*, *trnY-GUA-trnE-UUC*, *psbC-trnS-UGA*, *trnF-GAA-ndhJ*, *ccsA-ndhD*, and *rps15-ycf1-D2*. Reconstruction of the phylogenetic trees based on chloroplast genomes, strongly supported that *Aglaonema* was a sister to *Anchomanes*, and that the *Aglaonema* genus was classified into two sister clades including clade I and clade II, which corresponded to two sections, *Aglaonema* and *Chamaecaulon*, respectively. One variety and five cultivars, including *A. commutatum* ‘San Remo’, ‘Kai Sa’, ‘Pattaya Beauty’, ‘Silver Queen’, ‘Snow White’, and ‘White Horse Prince’, were classified into clade I; and the rest of the two cultivars, including ‘Sapphire’ and ‘White Gem’, were classified into clade II. Positive selection was observed in 34 protein-coding genes at the level of the amino acid sites among 77 chloroplast genomes of the Araceae family*.* Based on the highly variable regions and SSRs, 4 DNA markers were developed to differentiate the clade I and clade II in *Aglaonema*. In conclusion, this study provided chloroplast genomic resources for *Aglaonema*, which were useful for its classification and phylogeny.

## Introduction

*Aglaonema* is a herbaceous genus of the Araceae family, comprising 21 species, which are native to southeastern Asia from northeastern India across southern China and Indonesia through New Guinea^1^. *Aglaonema* species, commonly named Chinese evergreen, mostly inhabits humid and heavily shaded tropical forests^1,2^. The name *Aglaonema* means “shining stamen” in Greek. Based on its morphology, the *Aglaonema* genus is classified into two sections, which are *Chamaecaulon* and *Aglaonema*^1^. The section *Chamaecaulon* is characterized by a creeping and branching habit, exceedingly short petiolar sheaths, and the occurrence of cataphylls among the leaves^1^; and it includes *A. brevispathum* and *A. costatum*. Among them, *A. costatum* has been popular in cultivation for its variegated forms that have been introduced from the wild. For the section *Aglaonema*, the roots are rarely approaching the stem in thickness, and the stems are erect to partially decumbent; and it includes the rest of the species, such as *A. modestum*, *A. crispum*, *A. commutatum*, and *A. nitidum*. These four species have been cultivated in China and other Asian countries for centuries as indoor ornamental foliage plants or houseplants^2^. Additionally, *A. modestum* is a kind of traditional Chinese medicine, where the whole plant can be used as medicine, and it is successful in treating snakebite, sore throat, furuncle, and hemorrhoids^3,4^. Hence, *Aglaonema* plants not only have important ornamental and economic values, but also have high medicinal values. However, many cultivated cultivars and varieties of *Aglaonema* are derived from hybridization, natural mutation, and tissue-cultured mutation selection^5–8^, and the genetic relationships among their varieties and cultivars remain elusive. Therefore, it is difficult to identify between them based on only leaf morphology.

In the past decade, with the rapid development of high-throughput sequencing methods, the chloroplast genome sequencing has become lower in cost and higher quality than before. As the chloroplast genome is much smaller than the nuclear genome, and its coding genes are highly conservative^9^, it has been extensively used for studies on phylogenetic relationships in higher plants^10–30^. Chloroplast genomes can also be used as molecular markers for identifying species and cultivars, for examples, the chloroplast *trnL*-*trnF* intergenic spacer was used for molecular phylogenetic relationships between Lemnaceae and Araceae^21^; chloroplast *rps12* gene was applied in phylogenetic analysis in ferns^22^; and chloroplast genome was used as a super-barcode for identification of three cultivated varieties of *Scutellaria baicalensis*^26^. In the *Aglaonema* genus, the first complete chloroplast genome of *A. costatum* was published in 2020^13^. Two years later, six other complete chloroplast genomes from *A. modestum* and five variegated cultivars of *Aglaonema*, including ‘Hong Jian’, ‘Hong Yan’, ‘Lady Valentine’, ‘Red Valentine’ and ‘Red Vein’, were released^8^. *A. commutatum* was a complex and variable species because it had great variability in its leaf characteristics^1^. Therefore, it was difficult to solve the taxonomic problem of *A. commutatum* with only leaf morphology^1^. Although some phylogenetic studies have been done on *Aglaonema* species and cultivars^2,8,13^, the accurate genetic relationships among *A. commutatum* and popular green cultivars of *Aglaonema* in southern China still remain unknown. Due to lack of their chloroplast genomes information, their phylogenetic relationships have not been reported so far. Furthermore, there are no studies on developing reliable DNA markers in the *Aglaonema* genus based on complete chloroplast genomes. Therefore, it is essential to reconstruct phylogenetic relationships of *Aglaonema*, including more species and cultivars, and to develop reliable chloroplast DNA markers. This is not only useful for the conservation and utilization of *Aglaonema* germplasm resources, but will also spur the detection of new cultivars derived from breeding.

**Figure 1:**
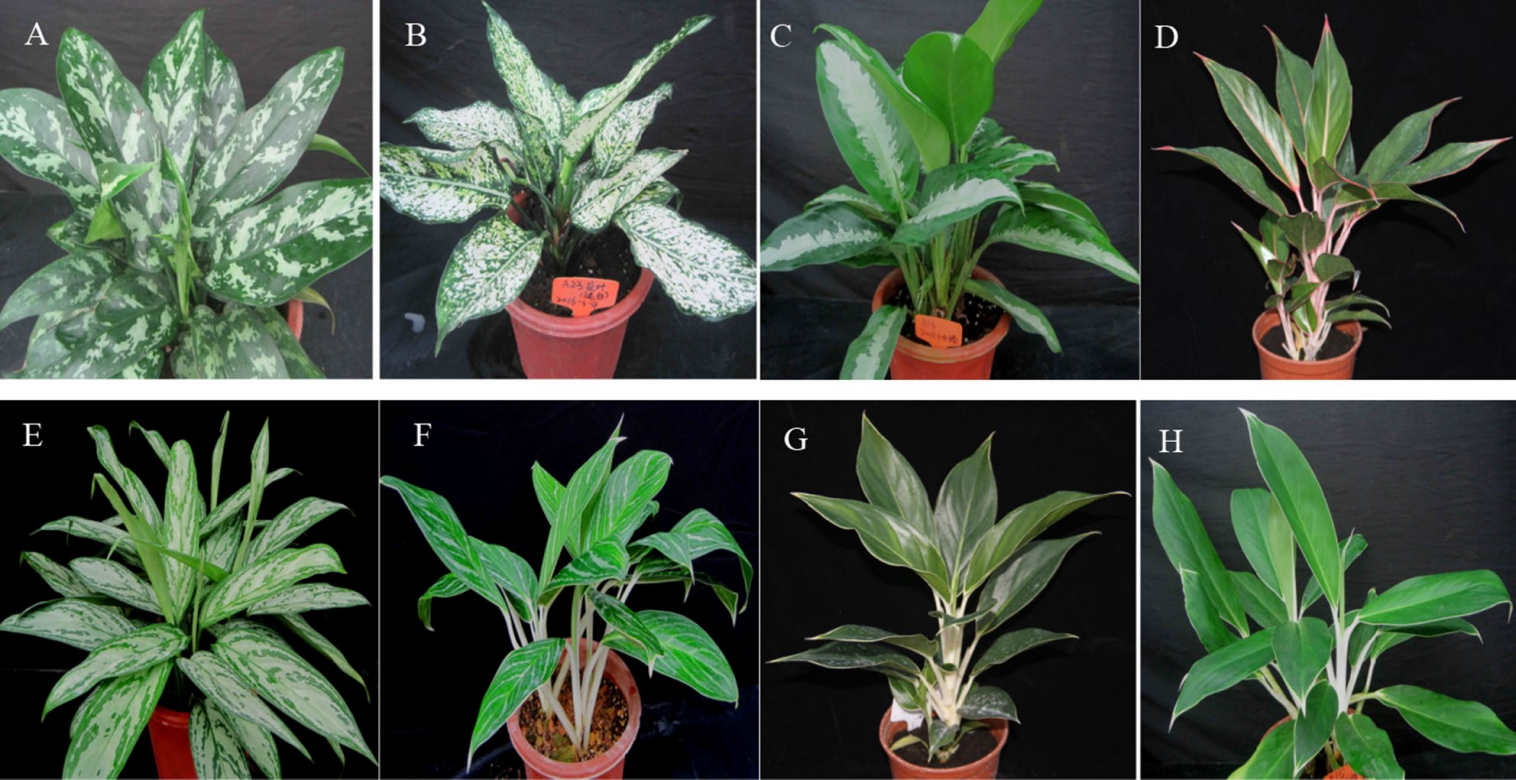
Leaf morphologies among *Aglaonema commutatum* and seven green cultivars of *Aglaonema*. (**A**) *Aglaonema commutatum* ‘San Remo’*,* (**B**) *Aglaonema* ‘Kai Sa’, (**C**) *Aglaonema* ‘Pattaya Beauty’, (**D**) *Aglaonema* ‘Sapphire’, (**E**) *Aglaonema* ‘Silver Queen’, (**F**) *Aglaonema* ‘Snow White’, (**G**) *Aglaonema* ‘White Gem’ and (**H**) *Aglaonema* ‘White Horse Prince’.

In this study, we sequenced the complete chloroplast genomes from one variety of *A. commutatum* and seven popular green cultivars of *Aglaonema*, namely, *A. commutatum* ‘San Remo’*,* ‘Kai Sa’, ‘Pattaya Beauty’, ‘Sapphire’, ‘Silver Queen’, ‘Snow White’, ‘White Gem’ and ‘White Horse Prince’ (Fig. 1). Then, we comprehensively compared them with seven previously reported chloroplast genomes of *Aglaonema* from NCBI. The main aims of this study were to: (1) supply newly sequenced complete chloroplast genomes for the *Aglaonema* genus and understand their overall genomes structures; (2) compare these 15 complete chloroplast genomes and identify highly divergent regions for the *Aglaonema* genus; (3) reconstruct phylogeny in the *Aglaonema* genus and Araceae family using complete chloroplast genomes, and examine the consistency with morphology taxonomy in the *Aglaonema* genus; and (4) develop novel DNA markers to discriminate *Aglaonema* species and cultivars.

## Results

### Characteristics of the 8 newly sequenced complete chloroplast genomes of *Aglaonema*

**Figure 2:**
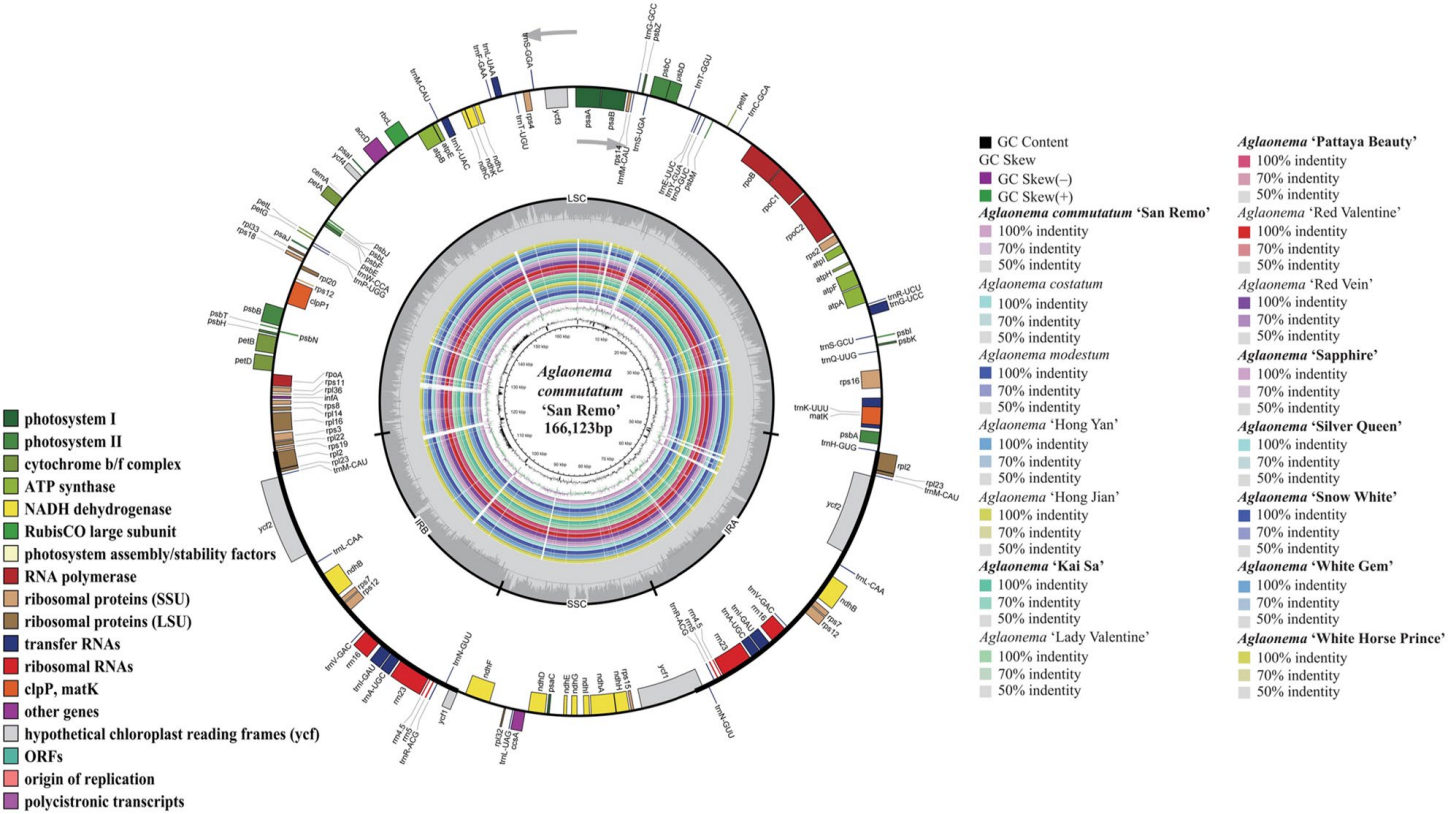
Chloroplast genome map of *A. commutatum* ‘San Remo’ (the outermost three rings) and CGView comparison of 15 *Aglaonema* chloroplast genomes (the inter rings with different colours). Genes belonging to different functional groups are shown in different colours in the outermost first ring. Genes shown on the outside of the outermost first ring are transcribed counter-clockwise and on the inside clockwise. Gray arrowheads indicate the direction of the genes. The tRNA genes are indicated by a one-letter code of amino acids with anticodons. LSC, large single-copy region; SSC, small single-copy region; and IR, inverted repeat. The innermost first black ring indicates the chloroplast genome size of *A. commutatum* ‘San Remo’. The innermost second and third rings indicate GC content and GC skew deviations in the chloroplast genome of *A. commutatum* ‘San Remo’, respectively: GC skew + indicates G > C, and GC skew–indicates G < C. From the innermost fourth color ring to the outwards 18th ring in turn: *A. commutatum* ‘San Remo’, *A. costatum*, *A. modestum*, *Aglaonema* ‘Hong Yan’, *Aglaonema* ‘Hong Jian’, *Aglaonema* ‘Kai Sa’, *Aglaonema* ‘Lady Valentine’, *Aglaonema* ‘Pattaya Beauty’, *Aglaonema* ‘Red Valentine’, *Aglaonema* ‘Red Vein’, *Aglaonema* ‘Sapphire’, *Aglaonema* ‘Silver Queen’, *Aglaonema* ‘Snow White’, *Aglaonema* ‘White Gem’, and *Aglaonema* ‘White Horse Prince’; chloroplast genome similar and highly divergent locations are represented by continuous and interrupted track lines, respectively. The 8 newly sequenced *Aglaonema* chloroplast genomes in this study are in bold.

The 8 newly sequenced *Aglaonema* plants, including *A. commutatum* ‘San Remo’*,* ‘Kai Sa’, ‘Pattaya Beauty’, ‘Sapphire’, ‘Silver Queen’, ‘Snow White’, ‘White Gem’, and ‘White Horse Prince’ (Fig. 1), generated about 6.28–7.50 Gb clean data for each sample after removing adapters and low-quality data (Table S1). Chloroplast genome lengths for these 8 samples ranged from 164,789 to 166,123 bp (Fig. 2, Table 1). All the 8 sequenced genomes showed a typical quadripartite structure comprising a LSC region (90,799–91,486 bp) and an SSC region (20,508–21,137 bp) separated by two IR regions (IRa and IRb) (26,661–26,750 bp) (Fig. 2, Table 1). The overall GC contents of these 8 genomes varied from 35.76% to 35.91% (Table 1). The IR region had the highest GC content (41.56–41.69%), followed by the LSC region (33.94–34.10%), while the SSC region had the lowest GC content (28.81–29.32%) (Table 1). The GC content of the protein-coding regions changed slightly from 37.72% to 37.76%. The GC content at the first codon position (45.32–45.36%) was higher than that at the second (38.19–38.28%) and third (29.57–29.65%) positions in the protein-coding genes of these 8 genomes (Table 1). They were submitted to the GenBank with accession numbers OR068724–OR068731 (Table 1).

**Table 1:** Characteristics of the eight newly sequenced chloroplast genomes of *Aglaonema*.

Genome characteristics	*A. commutatum* ‘San Remo’	‘Kai Sa’	‘Pattaya Beauty’	‘Sapphire’	‘Silver Queen’	‘Snow White’	‘White Gem’	‘White Horse Prince’
Genome size (bp)	166,123	165,584	165,657	165,229	164,789	165,052	165,152	165,335
LSC length (bp)	91,486	91,182	91,286	90,799	90,893	91,156	90,844	90,916
SSC length (bp)	21,137	21,068	20,985	21,006	20,508	20,508	20,976	21,089
IR length (bp)	26,750	26,667	26,661	26,712	26,694	26,694	26,666	26,665
Total GC (%)	35.76	35.82	35.84	35.86	35.91	35.85	35.87	35.82
GC in LSC (%)	33.94	34.01	34.04	34.10	34.03	33.94	34.08	34.02
GC in SSC (%)	28.81	28.96	28.85	28.95	29.32	29.30	28.99	28.90
GC in IR (%)	41.61	41.63	41.69	41.56	41.63	41.63	41.63	41.63
GC in PCD (%)	37.73	37.74	37.76	37.73	37.72	37.73	37.73	37.72
GC in first position	45.32	45.33	45.36	45.35	45.36	45.36	45.35	45.35
GC in second position	38.25	38.28	38.28	38.19	38.23	38.23	38.19	38.23
GC in third position	29.62	29.60	29.65	29.64	29.57	29.58	29.64	29.58
GC in tRNA genes	53.09	53.23	53.13	53.20	53.2	53.12	53.20	53.43
GC in rRNA genes	55.06	55.06	55.04	55.02	55.06	55.06	55.02	55.06
Total genes (different)	131 (112)	131 (112)	131 (112)	131 (112)	131 (112)	131 (112)	131 (112)	131 (112)
PCD (different)	86 (79)	86 (79)	86 (79)	86 (79)	86 (79)	86 (79)	86 (79)	86 (79)
tRNAs (different)	37 (29)	37 (29)	37 (29)	37 (29)	37 (29)	37 (29)	37 (29)	37 (29)
rRNAs (different)	8 (4)	8 (4)	8 (4)	8 (4)	8 (4)	8 (4)	8 (4)	8 (4)
GenBank accession number	OR068727	OR068729	OR068726	OR068730	OR068725	OR068724	OR068731	OR068728

GC, guanine-cytosine; PCD, protein-coding genes; LSC, large single copy region; SSC, small single copy region; and IR, inverted repeat.

Among these 8 genomes, each genome contained 131 functional genes, which consisted of 86 protein-coding genes, 37 tRNA genes, and 8 rRNA genes, respectively (Table 1, Table S2). Each genome contained 112 different genes, comprising 79 protein-coding genes, 29 tRNA genes and 4 rRNA genes, respectively (Table 1, Table S2). There were 18 genes with two copies in the IR regions, including *ndhB*, *rpl2*, *rpl23*, *rps7*, *rps12*, *ycf1*, *ycf2*, *trnM-CAU*, *trnL-CAA*, *trnV-GAC*, *trnI-GAU*, *trnA-UGC*, *trnR-ACG*, *trnN-GUU*, *rrn4.5*, *rrn5*, *rrn16* and *rrn23* (Table 2, Table S2). Sixteen genes contained one intron, comprising *trnA-UGC*, *trnG-UCC*, *trnI-GAU*, *trnK-UUU*, *trnL-UAA*, *trnV-UAC*, *atpF*, *ndhA*, *ndhB*, *rpoC1*, *petB*, *petD*, *rpl2*, *rpl16*, *rps12*, and *rps16,* while *ycf3* and *clpP* each contained two introns (Table 2, Table S2).

**Table 2:** Genes present in the eight newly sequenced chloroplast genomes of *Aglaonema*.

Category	Function	Genes
Photosynthesis	Photosystem I	psaA, psaB, psaC, psaI, psaJ
	Photosystem II	psbA, psbB, psbC, psbD, psbE, psbF, psbH, psbI, psbJ, psbK, psbL, psbM, psbN, psbT, psbZ
	Cytochrome b/f	petA, petB*, petD*, petG, petL, petN
	ATP synthase	atpA, atpB, atpE, atpF*, atpH, atpI
	NADH dehydrogenase	ndhA*, ndhB (× 2)*, ndhC, ndhD, ndhE, ndhF, ndhG, ndhH, ndhI, ndhJ, ndhK
	Rubisco	rbcL
Self-replication	RNA polymerase	rpoA, rpoB, rpoC1*, rpoC2
	Large subunit ribosomal proteins	rpl2 (× 2)*, rpl14, rpl16*, rpl20, rpl22, rpl23 (× 2), rpl32, rpl33, rpl36
	Small subunit ribosomal proteins	rps2, rps3, rps4, rps7 (× 2), rps8, rps11, rps12 (× 2)*, rps14, rps15, rps16*, rps18, rps19 (× 2)
	Ribosomal RNAs	*rrn4.5* (× 2), *rrn5* (× 2), *rrn16* (× 2), *rrn23* (× 2)
	Transfer RNAs	trnA-UGC (× 2)*, trnC-GCA, trnD-GUC, trnE-UUC, trnF-GAA, trnfM-CAU, trnG-GCC, trnG-UCC*, trnH-GUG, trnI-GAU (× 2)*, trnK-UUU*, trnL-CAA (× 2), trnL-UAA*, trnL-UAG, trnM-CAU (× 3), trnN-GUU (× 2), trnP-UGG, trnQ-UUG, trnR-ACG (× 2), trnR-UCU, trnS-GCU, trnS-GGA, trnS-UGA, trnT-GGU, trnT-UGU, trnV-GAC (× 2), trnV-UAC*, trnW-CCA, trnY-GUA
Others	Other proteins	accD, ccsA, cemA, clpP**, infA, matK
	Proteins of unknown function	*ycf1* (× 2), *ycf2* (× 2), ycf3**, *ycf4*

× 2, gene with two copies; × 3, gene with three copies; *, each gene containing only one intron; and **, each gene containing two introns.

### Codon usage analysis

**Figure 3:**
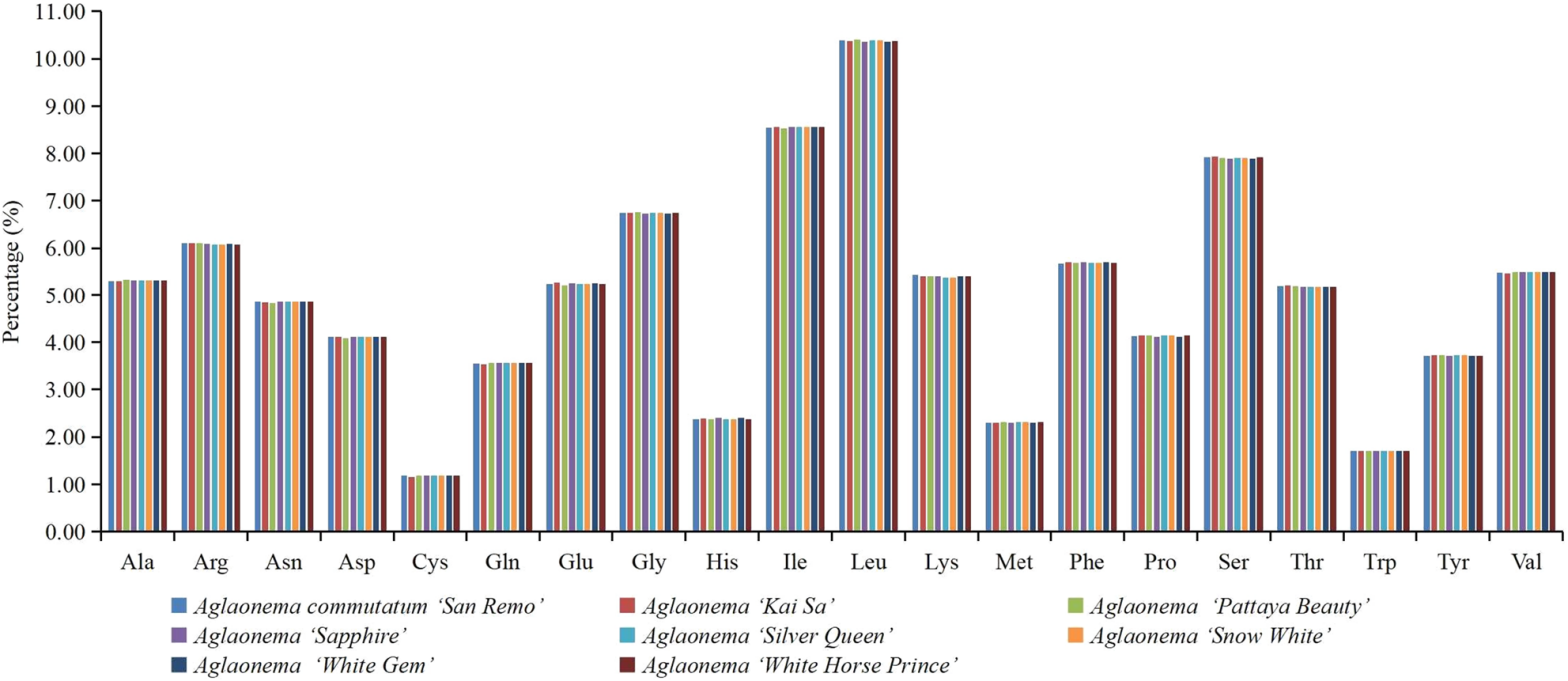
Codon content of 20 amino acids of all protein-coding genes in the 8 newly sequenced *Aglaonema* chloroplast genomes.

The codon usage of the 8 sequenced *Aglaonema* chloroplast genomes is shown in Table S3. Protein-coding genes contained 26,259 codons to 26,353 codons among these 8 genomes (Table S3). Among these codons, those for leucine and isoleucine were the first and second most common in these 8 genomes (Fig. 3, Table S3). The use of the codons ATG and TGG, encoding Met and Trp respectively, exhibited no bias (RSCU = 1.00) in these 8 *Aglaonema* chloroplast genomes (Fig. 3, Table S3). The codons with the three highest RSCU values (AGA, GCT, and TTA) and the four lowest RSCU values (AGC, GGC, CGC, and CTG) were found in the protein-coding genes codons of these 8 genomes (Table S3). Additionally, 29 codons with RSCU higher than 1.00 were A/T-ending codons and only one codon with RSCU higher than 1.00 was G/C-ending (Table S3). The results of RSCU > 1.00 indicated that these 8 *Aglaonema* genomes had a higher usage frequency for A/T-ending than G/C-ending.

### Analyses of long repeats and SSRs

**Figure 4:**
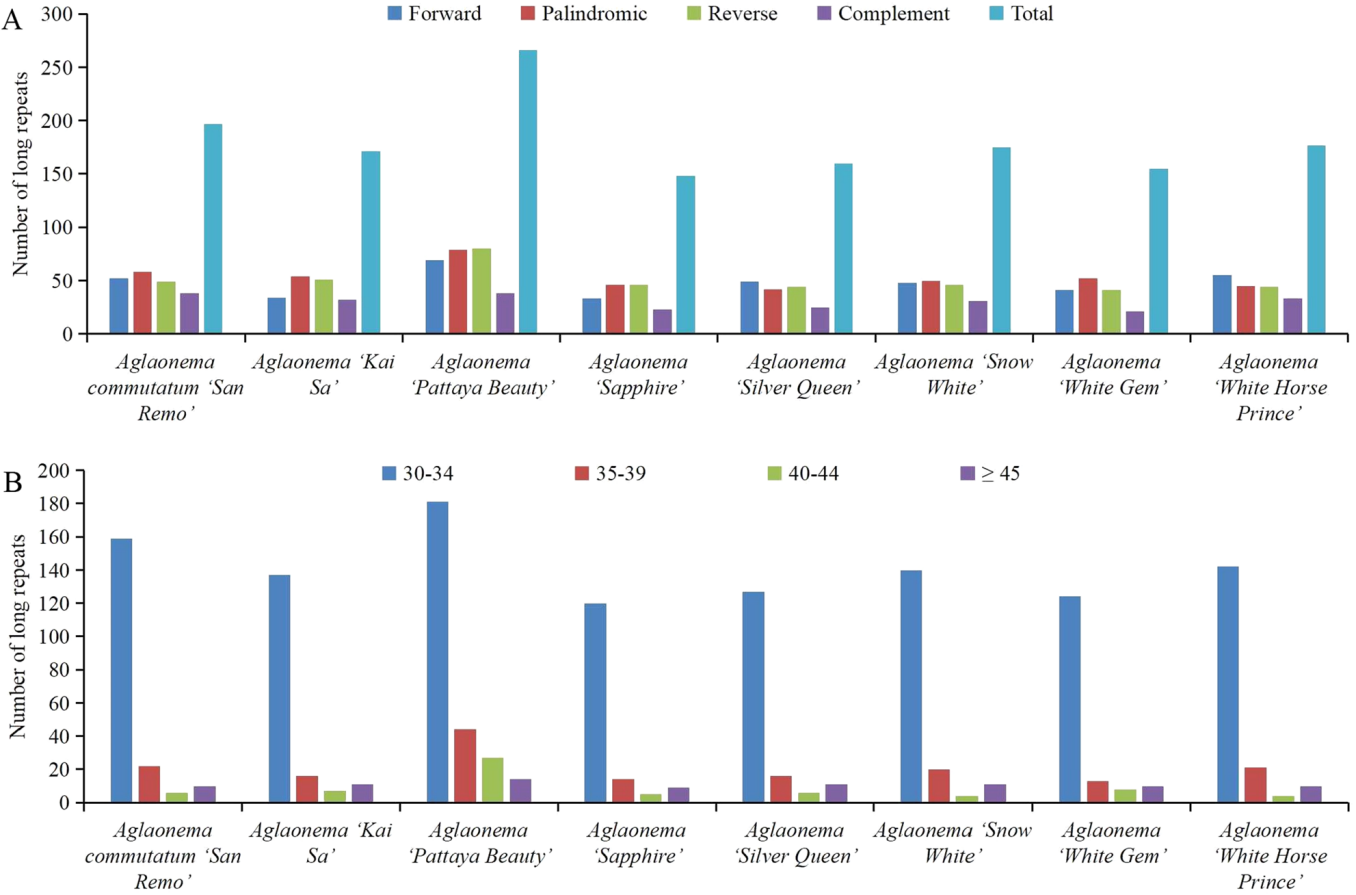
Long repeat sequences distribution in the 8 newly sequenced *Aglaonema* chloroplast genomes. (**A**) Total number of four long repeat types. (**B**) Length distribution of long repeats in each sequenced chloroplast genome.

Long repeats of the 8 newly sequenced genomes were analyzed by REPuter and the results are displayed in Fig. 4 and Table S4. Among these 8 genomes, ‘Pattaya Beauty’ had the largest number (266), and ‘Sapphire’ had the smallest number (148) of long repeats (Fig. 4, Table S4). Four different types of long repeats were found, including forward, palindromic, reverse, and complement repeats. The number of forward repeats varied from 33 to 69, the number of palindromic repeats varied from 42 to 79, the number of reverse repeats varied from 41 to 80, and the number of complement repeats varied from 21 to 38 (Fig. 4A, Table S4). The length of long repeats varied among these 8 genomes (Fig. 4B, Table S4). Long repeats with lengths of 30–34 bp were found to be the most common in these 8 genomes, followed by 35–39 bp (Fig. 4B, Table S4).

**Figure 5:**
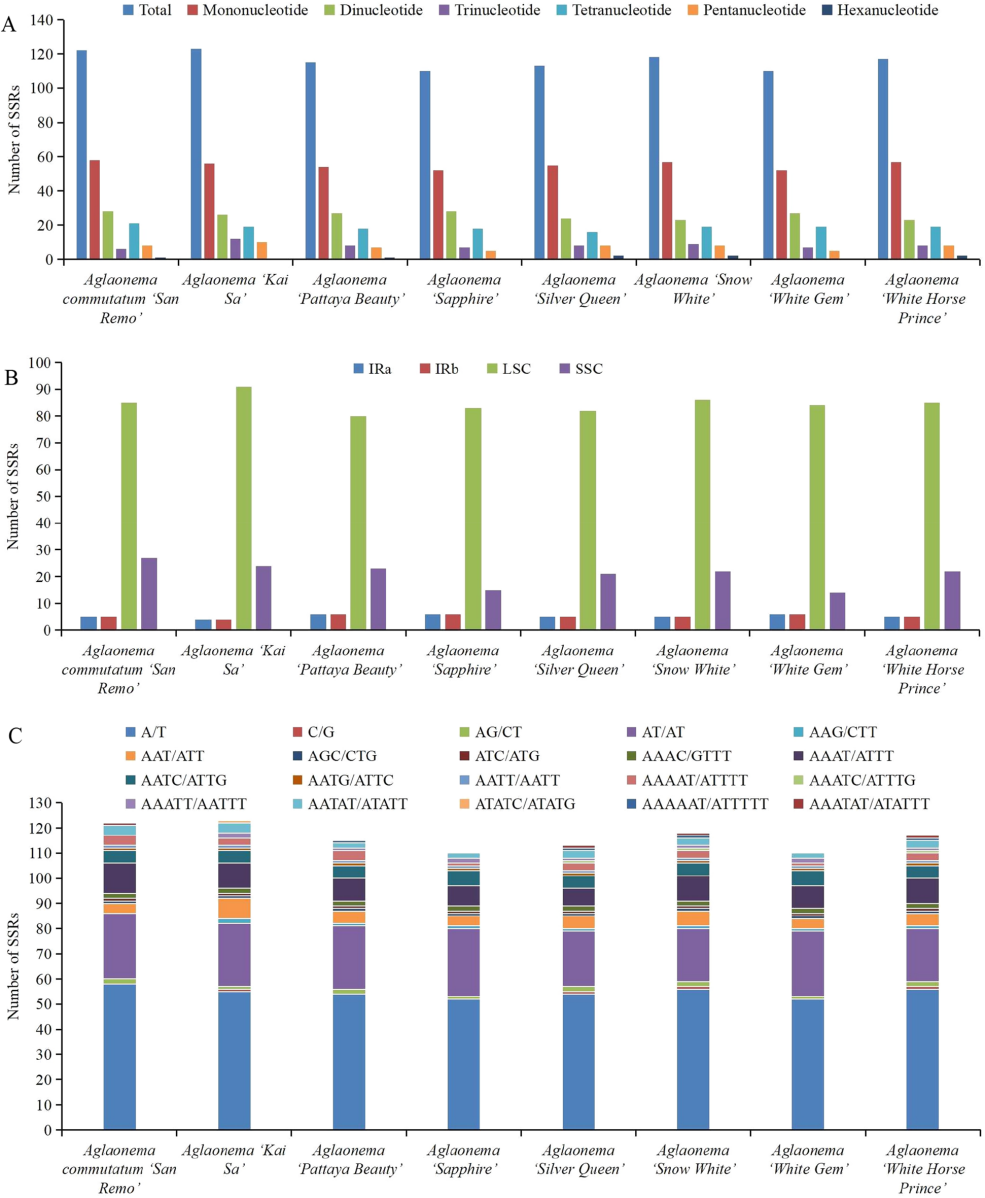
Distribution of SSRs in the 8 newly sequenced *Aglaonema* chloroplast genomes. (**A**) Number of different SSR types. (**B**) Frequency of the identified SSRs in the LSC, SSC and IR regions. (**C**) Frequency of the identified SSRs in different repeat class types.

SSRs in the 8 newly sequenced genomes were also analyzed. The number of SSRs ranged from 110 to 123 (Fig. 5A, Table S5). Mononucleotide SSRs were the most abundant with numbers ranging from 52 to 58, followed by dinucleotide SSRs ranging from 23 to 28, tetranucleotide SSRs ranging from 16 to 21, trinucleotide SSRs ranging from 6 to 12, pentanucleotide SSRs ranging from 5 to 10, and hexanucleotide SSRs ranging from 0 to 2 (Fig. 5A, Table S5). It is interesting to find that hexanucleotide SSRs were not found in three cultivars, namely, ‘Kai Sa’, ‘Sapphire’ and ‘White Gem’ (Fig. 5A, Table S5). SSRs were more frequently located in the LSC regions (80–91 loci) than in the SSC regions (14–27 loci) and IR regions (4–6 loci) of these 8 genomes (Fig. 5B, Table S5). Additionally, among these 8 genomes, most of the mononucleotide SSRs were A/T repeats, with numbers ranging from 52 to 58 (Fig. 5C, Table S5). In the dinucleotide repeats, the AT/AT repeats were observed most frequently, with numbers ranging from 21 to 27 (Fig. 5C, Table S5). In the trinucleotide repeats, the AAT/ATT repeats were the richest type, with numbers ranging from 4 to 8 (Fig. 5C, Table S5). In the tetranucleotide category, the AAAT/ATTT repeats were the most abundant type, with numbers ranging from 7 to 12, followed by AATC/ATTG with numbers ranging from 5 to 6 (Fig. 5C, Table S5).

### Contraction and expansion of IRs

A comprehensive comparison for four borders, LSC/IRa, LSC/IRb, SSC/IRa and SSC/IRb, was performed among the 8 newly sequenced chloroplast genomes of *Aglaonema* (Fig. S1). Regarding IRa/LSC borders, the *rpl2, trnH-GUG* and *psbA* genes were located at the IRa/LSC borders in these 8 genomes. The ends of *trnH-GUG* were just located at the borders of IRa/LSC, and the distances between the ends of *rpl2* and IRa/LSC borders ranged from 52 to 62 bp (Fig. S1). The *psbA* genes were all located at the LSC regions, with distances ranging from 565 to 601 bp from the IRa/LSC borders (Fig. S1). Among these 8 chloroplast genomes of *Aglaonema*, the *rps19* and *rpl2* genes were located at the LSC/IRb borders, respectively (Fig. S1). There were 21–22 bp distances between the ends of *rps19* and the LSC/IRb borders among these 8 genomes, and the distances between the starts of *rpl2* and the LSC/IRb borders ranged from 53 to 63 bp (Fig. S1). The SSC/IRa border was located in the *ycf1* coding region, which expanded into the IRa regions with lengths ranging from 733 to 772 bp (Fig. S1). The IRb/SSC borders of these 8 genomes, were all located in *ycf1*, and *ycf1* expanded into the SSC regions by 10 bp to 41 bp, respectively (Fig. S1). The distances of the starts of *ndhF* and the IRb/SSC borders ranged from 331 to 719 bp (Fig. S1). In conclusion, the IR/SC borders of these 8 *Aglaonema* chloroplast genomes were highly conserved.

### Analyses of SNPs and indels

First, using the chloroplast genome of *A. commutatum* ‘San Remo’ as the reference, SNP/indel loci of the chloroplast genomes of ‘Kai Sa’, ‘Pattaya Beauty’, ‘Sapphire’, ‘Silver Queen’, ‘Snow White’, ‘White Gem’, and ‘White Horse Prince’ were detected. Regarding ‘Kai Sa’ versus *A. commutatum* ‘San Remo’, 228 protein-coding gene SNPs, 356 intergenic SNPs, and 133 indels were identified (Table 3, Tables S6 and S7), and the lengths of indels were mainly between 1 and 6 bp (Fig. S2). Concerning ‘Pattaya Beauty’ versus *A. commutatum* ‘San Remo’, 85 protein-coding gene SNPs, 141 intergenic SNPs, and 60 indels were detected; for ‘Sapphire’ versus *A. commutatum* ‘San Remo’, 346 protein-coding gene SNPs, 498 intergenic SNPs, and 150 indels were identified; for ‘Silver Queen’ versus *A. commutatum* ‘San Remo’, 140 protein-coding gene SNPs, 175 intergenic SNPs, and 84 indels were identified; for ‘Snow White’ versus *A. commutatum* ‘San Remo’, 139 protein-coding gene SNPs, 177 intergenic SNPs, and 86 indels were identified; for ‘White Gem’ versus *A. commutatum* ‘San Remo’, 346 protein-coding gene SNPs, 506 intergenic SNPs, and 150 indels were identified; and for ‘White Horse Prince’ and *A. commutatum* ‘San Remo’, 139 protein-coding gene SNPs, 179 intergenic SNPs, and 83 indels were identified (Table 3, Tables S6 and S7, Fig. S2).

**Table 3:** SNPs and indels among the eight newly sequenced chloroplast genomes of *Aglaonema*.

Comparison pairs	Protein-coding genes SNPs	Intergenic regions SNPs	Total SNPs	Insertions	Deletions	Indels
‘Kai Sa’ versus* A. commutatum* ‘San Remo’	228	356	584	69	64	133
‘Pattaya Beauty’ versus* A. commutatum* ‘San Remo’	85	141	226	33	27	60
‘Sapphire’ versus* A. commutatum* ‘San Remo’	346	498	844	83	67	150
*‘*Silver Queen’ versus* A. commutatum* ‘San Remo’	140	175	315	46	38	84
‘Snow White’ versus* A. commutatum* ‘San Remo’	139	177	316	46	40	86
‘White Gem’ versus* A. commutatum* ‘San Remo’	346	506	852	83	67	150
‘White Horse Prince’ versus* A. commutatum* ‘San Remo’	139	179	318	45	38	83
‘Kai Sa’ versus ‘White Gem’	284	424	708	80	80	160
‘Pattaya Beauty’ versus ‘White Gem’	353	515	868	72	84	156
‘Sapphire’ versus ‘White Gem’	0	1	1	1	0	1
*‘*Silver Queen’ versus ‘White Gem’	313	437	750	72	79	151
‘Snow White’ versus ‘White Gem’	312	441	753	72	79	151
‘White Horse Prince’ versus ‘White Gem’	333	436	769	73	79	152

Second, the chloroplast genomes of seven green cultivars of *Aglaonema*, namely, ‘Kai Sa’, ‘Pattaya Beauty’, ‘Sapphire’, ‘Silver Queen’, ‘Snow White’, and ‘White Horse Prince’, were also analyzed to detect SNPs/indels, respectively, using the chloroplast genome of ‘White Gem’ as the reference. Concerning ‘Kai Sa’ versus ‘White Gem’, 284 protein-coding gene SNPs, 424 intergenic SNPs, and 160 indels were identified; concerning ‘Pattaya Beauty’ versus ‘White Gem’, 353 protein-coding gene SNPs, 515 intergenic SNPs, and 156 indels were identified; concerning ‘Silver Queen’ versus ‘White Gem’, 313 protein-coding gene SNPs, 437 intergenic SNPs, and 151 indels were identified; concerning ‘Snow White’ versus ‘White Gem’, 312 protein-coding gene SNPs, 441 intergenic SNPs, and 151 indels were identified; and concerning ‘White Horse Prince’ versus ‘White Gem’, 333 protein-coding gene SNPs, 436 intergenic SNPs, and 152 indels were identified (Table 3, Tables S6 and S7, Fig. S2). Finally, for ‘Sapphire’ versus ‘White Gem’, only 1 intergenic SNP and 1 indel were identified (Table 3, Tables S6 and S7, Fig. S2).

### Comparative chloroplast genomics in the *Aglaonema* genus

**Figure 6:**
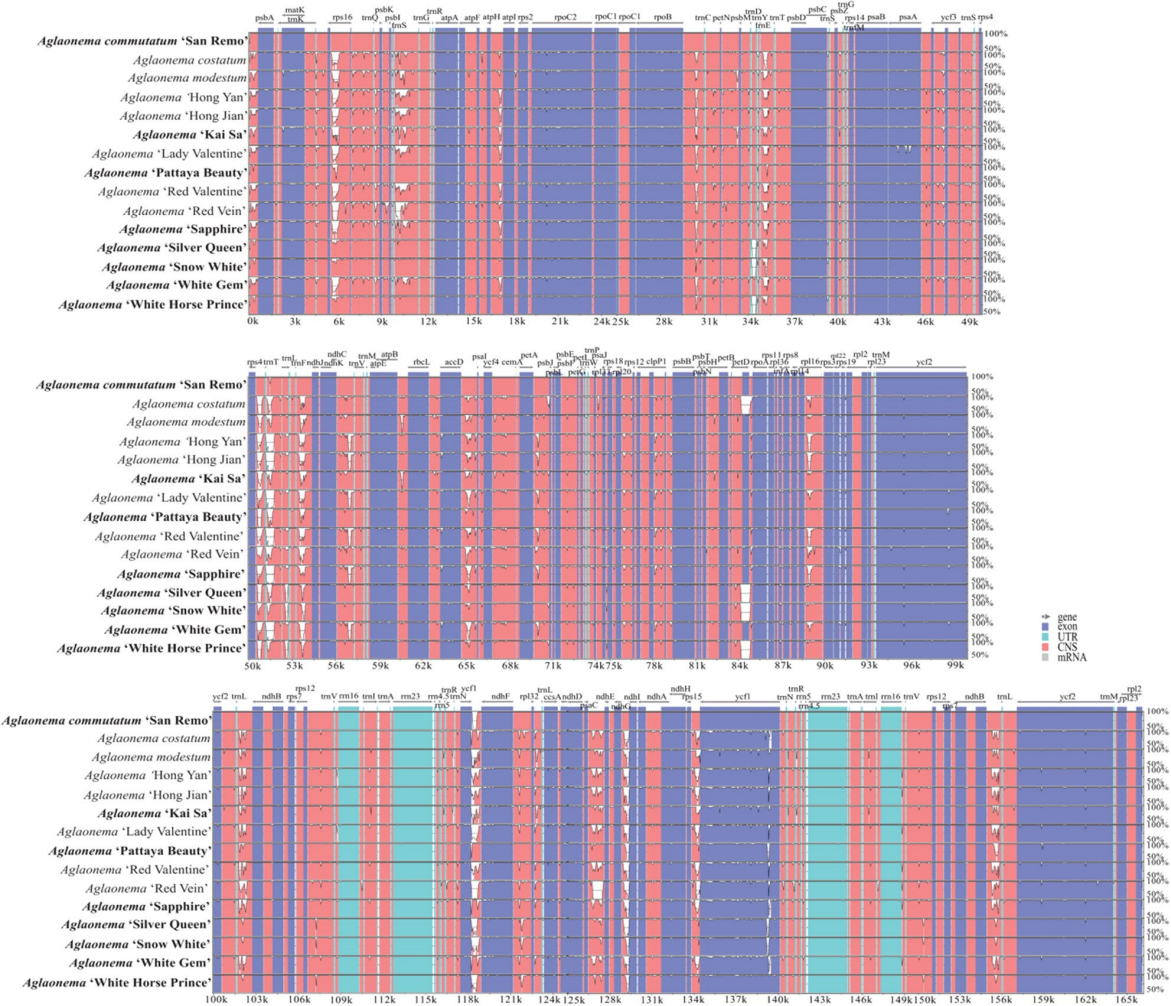
Chloroplast genome comparison of 15 *Aglaonema* chloroplast genomes using *A. commutatum* ‘San Remo’ as the reference. Gray arrows and thick black lines above the alignment indicate gene orientation. Purple bars represent exons, sky-blue bars represent untranslated regions (UTRs), red bars represent non-coding sequences (CNS), gray bars represent mRNA and white regions represent sequence differences among analyzed chloroplast genomes. The y-axis represents the identity percentage ranging from 50 to 100%. The 8 newly sequenced *Aglaonema* chloroplast genomes in this study are in bold.

Using the complete chloroplast genome of *A. commutatum* ‘San Remo’ as the reference, a comparative analysis based on mVISTA program was performed among 15 complete chloroplast genomes of *Aglaonema*, which included the 8 newly sequenced ones and 7 published ones from NCBI (Fig. 6). The results indicated that the LSC and SSC regions were more divergent than the two IR regions (Fig. 6). In the protein-coding regions, most protein-coding genes were highly conserved except for *rps16*, *trnS*, *trnE*, *rpl32*, *trnV* and *ycf1* (Fig. 6). The highly divergent regions among these 15 genomes mainly located in the intergenic regions, including *trnH*-*psbA*, *trnS*-*trnG*, *trnY-trnE* and *trnF-ndhJ* in LSC region as well as *ndhF-rpl32*, *ccsA-ndhD*, and *rps15*-*ycf1* in SSC region (Fig. 6). The CGview result also revealed that the IR regions were less divergent than the LSC and SSC regions (innermost 4th colour ring to outwards 18th ring in Fig. 2). In comparison to the chloroplast genome of *A. commutatum ‘*San Remo*’* (innermost 4th colour ring in Fig. 2), the rest of the 14 chloroplast genomes showed four divergent regions in LSC (*trnS-trnG*, *trnY-trnE*, *psbC-trnS*, and *trnF-ndhJ*), two divergent regions in SSC (*ccsA-ndhD* and *rps15*-*ycf1*) and one divergent region in IRa (*rpl22-rps19*).

**Figure 7:**
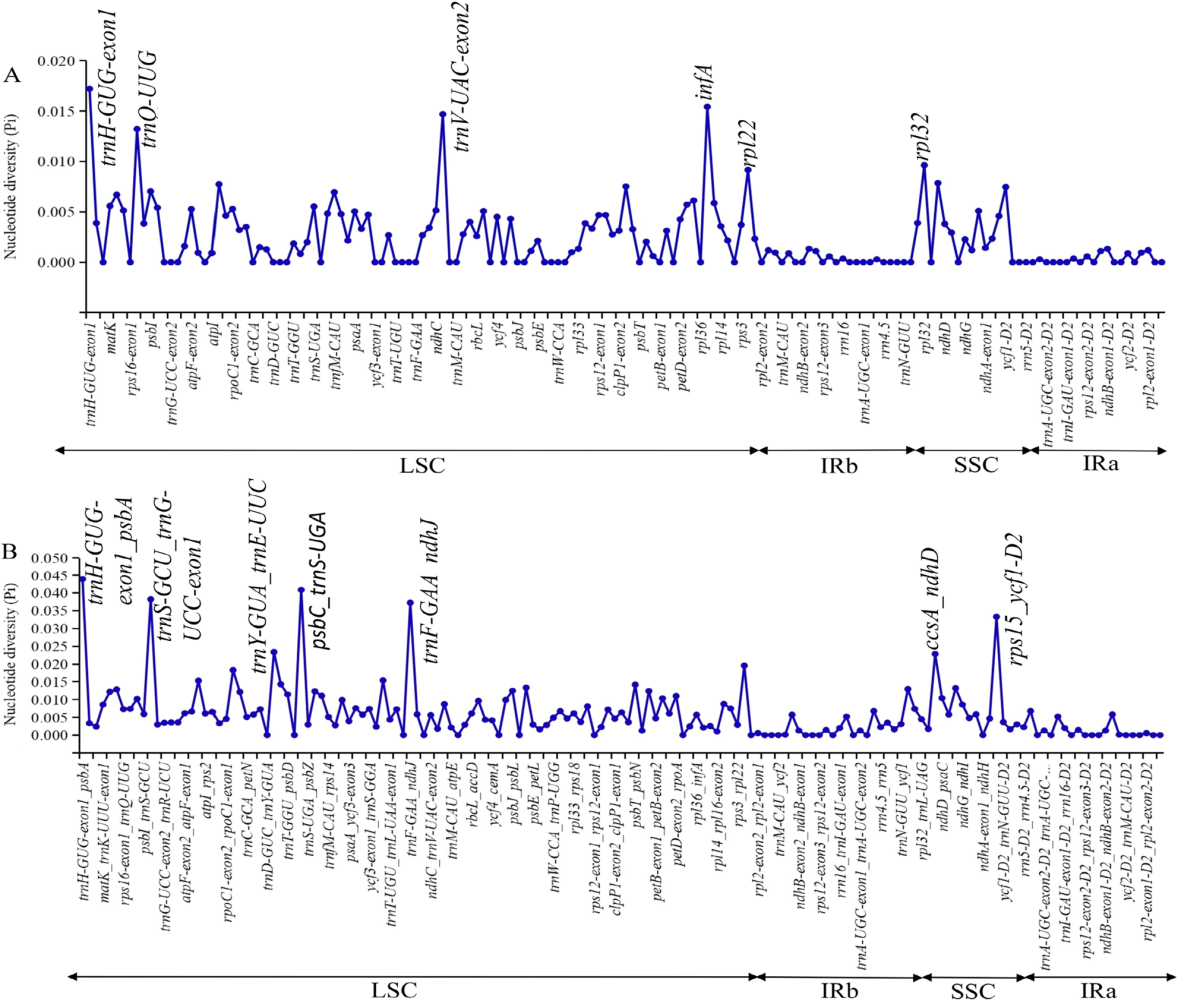
Comparisons of nucleotide diversity (Pi) values among 15 complete chloroplast genomes of the genus *Aglaonema*. (**A**) Protein-coding genes. Protein-coding genes with Pi values > 0.009 are labelled with gene names. (**B**) Intergenic regions. Intergenic regions with Pi values > 0.022 are labelled with intergenic region names.

Nucleotide diversity (Pi) and single nucleotide substitutions in the LSC, SSC, IRa, IRb and the total chloroplast genomes among 15 complete chloroplast genomes of *Aglaonema* were analyzed (Fig. 7, Table S8). Concerning the protein-coding regions, Pi values for each gene ranged from 0 to 0.0177, and the average value was 0.0023. The *trnH-GUG-exon1* had the highest Pi value (0.0177) followed by the other four gene regions of *trnV-UAV-exon2*, *infA*, *rpl22*, and *rpl32* (Pi > 0.0091) (Fig. 7A). For the intergenic regions, Pi values ranged from 0 to 0.0439 (*trnH-GUG-exon1-psbA*) and had an average of 0.006. Seven of these intergenic regions also showed remarkably high values (Pi > 0.0228), including *trnH-GUG-exon1-psbA*, *trnS-GCU-trnG-UCC-exon1*, *trnY-GUA-trnE-UUC*, *psbC-trnS-UGA*, *trnF-GAA-ndhJ*, *ccsA-ndhD*, and *rps15-ycf1-D2* (Fig. 7B). Additionally, 15 complete chloroplast genomes of *Aglaonema* were aligned with a matrix of 166,123 bp with 2,431 variable sites (1.46%) and 2,387 parsimony informative sites (1.43%). The Pi value of the complete chloroplast genome was 0.0047 (Table S8). The SSC region had the highest Pi value (0.0065) and the IRb region had the lowest Pi value (0.0011) (Table S8). By using region length > 100 bp and combining the results of Pi > 0.022, CGView and mVISTA for the selection of potential molecular markers for *Aglaonema*, 7 regions were found: *trnH-GUG-exon1-psbA*, *trnS-GCU-trnG-UCC-exon1*, *trnY-GUA-trnE-UUC*, *psbC-trnS-UGA*, *trnF-GAA-ndhJ*, *ccsA-ndhD*, and *rps15-ycf1-D2* (Table S8).

### Phylogenetic relationships in the *Aglaonema* genus and Araceae family

**Figure 8:**
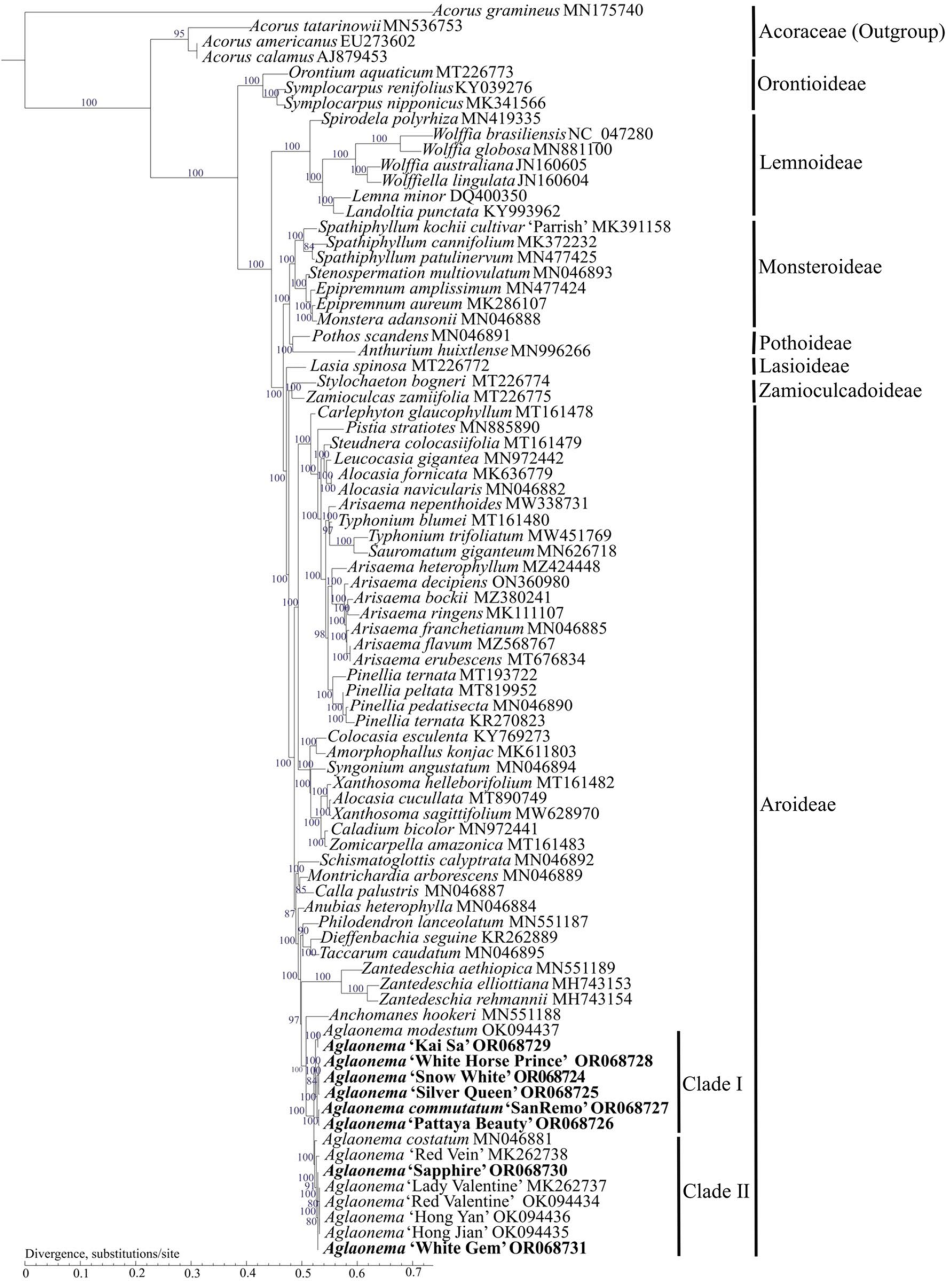
Phylogenetic tree of 77 complete chloroplast genomes of the Araceae family using the ML method. The 8 newly sequenced *Aglaonema* chloroplast genomes in this study are in bold.

To study the phylogenetic relationships of *A. commutatum* ‘San Remo’ and the seven green cultivars of *Aglaonema* within the Araceae family, two phylogenetic trees were constructed using the complete chloroplast genomes by Maximum likelihood (ML) and Bayesian inference (BI) methods, respectively (Fig. 8, Fig. S3). The species of Acoraceae were used as outgroups. Both ML and BI trees displayed the same topological structures (Fig. 8, Fig. S3). In this study, we used the same criteria as previously reported^23^, which defined strong support as 85% ≤ ML bootstrap (MLBS) ≤ 100% and 0.90 ≤ BI posterior probability (BIPP) ≤ 1.0; moderate support as 70% ≤ MLBS < 85% and 0.80 ≤ BIPP < 0.90; and weak support as MLBS < 70% and BIPP < 0.80. The analyzed Araceae species were divided into seven subfamilies, including Aroideae, Lasioideae, Lemnoideae, Monsteroideae, Orontioideae, Pothoideae, and Zamioculcadoideae with strongly supported values (BS = 100% for the ML tree and PP = 1 for the BI tree nodes) (Fig. 8, Fig. S3).

In both the phylogenetic trees, within the Aroideae subfamily, *Aglaonema* was a sister to *Anchomanes* with strong supports (BS = 100%, and PP = 1), and *Aglaonema* + *Anchomanes* and *Zantedeschia* were strongly supported as sister genera (BS = 97%, and PP = 1) (Fig. 8, Fig. S3). Within the *Aglaonema* genus, there were two sister clades, including clade I and clade II with strong supports (BS = 100%, and PP = 1) (Fig. 8, Fig. S3). Within clade I, *A. commutatum* ‘San Remo’ and ‘Pattaya Beauty’ were clustered together, forming one cluster with strong supports (BS = 100%; and PP = 1); ‘Snow White’, *‘*Silver Queen’, and ‘White Horse Prince’ were clustered one by one, forming another cluster with moderate to strong supports (BS = 84 − 100%; and PP = 0.99 − 1); then the two clusters were sister to the cluster three including *A. modestum* and ‘Kai Sa’, with strong supports (BS = 100%; and PP = 1) (Fig. 8, Fig. S3). Within clade II, ‘Hong Jian’, ‘Hong Yan’, ‘Red Valentine’ and ‘Lady Valentine’ were clustered together, forming one cluster; then the cluster, ‘White Gem’, ‘Sapphire’, ‘Red Vein’ and *A. costatum* were clustered one by one with weak/moderate to strong supports (BS = 80–100%; and PP = 0.61–1) (Fig. 8, Fig. S3).

### Selective pressure analyses in the Araceae family

The ratio (ω) of non-synonymous (dN) to synonymous (dS) substitution (dN/dS) for 61 shared protein-coding genes was analyzed among 77 complete chloroplast genomes of the Araceae family (Table S9). In this study, using the M8 model for estimating gene selection pressure, 34 protein-coding genes were under positive selection with a posterior probability greater than 0.95 using the BEB method (Table S10). These protein-coding genes with positive selection sites could be divided into five categories: subunits of photosystem (*psaB*, *psaC*, *psbA*, *psbB* and *psbC*), subunits of ATP synthase (*atpA*, *atpB*, *atpF* and *atpI*), subunits of NADH dehydrogenase (*ndhA*, *ndhB*, *ndhC*, *ndhF*, and *ndhH*), subunits of ribosome (*rpl2*, *rpl14*, *rpl16*, *rps3*, *rps7*, *rps8*, *rps11*, *rps15* and *rps18*) and others (*rpoA*, *rpoB*, *rpoC1*, *rpoC2*, *rbcL*, *ccsA*, *clpP*, *matK*, *ycf2*, *ycf3* and *ycf4*). Among these 34 protein-coding genes, *ycf2* harboured the highest number of positive amino acids sites (72), followed by *rpoC2* (23), *rbcL* (12), *matK* (7), *atpA* (6), *atpB* (5), *rpoB* (5) and *ndhF* (4); the remaining 26 protein-coding genes had one to three positive amino acids sites (Table S10).

### Molecular markers development based on the *Aglaonema* chloroplast genomes

**Figure 9:**
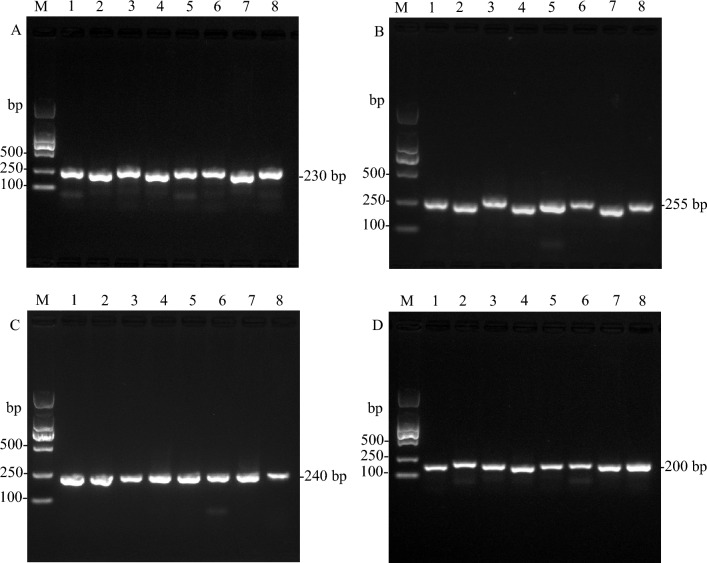
The PCR results of the amplification of DNA markers using designed primers. (**A**) Primer30. **(B)** Primer83. (**C**) Primer1. (**D**) Primer3. Lane M is the marker of DL2000. The lanes from 1 to 8 correspond to the products amplified from *A. commutatum* ‘San Remo’*,* ‘Kai Sa’, ‘Pattaya Beauty’, ‘Sapphire’, ‘Silver Queen’, ‘Snow White’, ‘White Gem’ and ‘White Horse Prince’, respectively.

To identify *A. commutatum* ‘San Remo’ and the seven green cultivars of *Aglaonema*, we selected several highly divergent regions and SSRs containing regions to develop the DNA markers. Finally, four markers could successfully discriminate some *Aglaonema* species and cultivars. In the present study, we only showed the results of the valid markers. There were two valid DNA markers, namely, Primer30 and Primer83, which were located in the two divergent regions *trnY-GUA-trnE-UUC* and *rps15-ycf1*, respectively (Table S11, Fig. 7). Additionally, there were two other valid markers, namely, Primer1 and Primer3, which contained SSRs and were located in the *psbA* and *trnK-UUU-exon1-rps16-exon2*, respectively (Table S11). These four markers were used to differentiate ‘Sapphire’ and ‘White Gem’ from *A. commutatum* ‘San Remo’, ‘Kai Sa’, ‘Pattaya Beauty’, *‘*Silver Queen’, ‘Snow White’, and ‘White Horse Prince’ (Fig. 9, Fig. S4). Based on the results of the two phylogenetic trees, *A. commutatum* ‘San Remo’, ‘Kai Sa’, ‘Pattaya Beauty’, *‘*Silver Queen’, ‘Snow White’, and ‘White Horse Prince’ were clustered into clade I, and ‘Sapphire’ and ‘White Gem’ were clustered into clade II (Fig. 8, Fig. S3). The results of these four DNA markers were consistent with the results of the phylogenetic trees, which could be used for further studies on identification of *Aglaonema* species and cultivars.

## Discussion

In this study, 8 complete chloroplast genomes from *Aglaonema* in the Araceae family, were sequenced, assembled and performed for their comparative genomics with other related *Aglaonema* species and cultivars^8,13^. All these 8 genomes possessed a typical quadripartite structure, as reported for other Araceae species, such as *A. modestum*^8^, *Epipremnum aureum*^11^, *A. costatum*^13^, *Anthurium huixtlense* and *Pothos scandens*^16^. These 8 genomes each encoded 112 different genes, including 79 protein-coding genes, 4 rRNA genes, and 29 tRNA genes (Table 1). This result was consistent with the protein-coding gene number, rRNA gene number and tRNA gene number in a previous report for *A. modestum*, ‘Red Valentine’, ‘Hong Yan’, and ‘Hong Jian’^8^. However, there were some variations in these 8 chloroplast genome lengths, with *A. commutatum* ‘San Remo’ having the longest genome length, which was 166,123 bp, and ‘Silver Queen’ being the shortest, at only 164,789 bp (Table 1). Notably, the chloroplast genomes lengths varied by 1.3 kb herein. This finding was also reported for the *Polystachya* species with about 3.8 kb differences in chloroplast genome lengths^19^. The reasons for genome length variations may be because of the number of genes or introns loss and gain, IR contraction and expansion, and variations of the intergenic regions^13,17,19,24^.

In many chloroplast genomes of higher plants, leucine and cysteine were identified as the most common and the least common codons, respectively^8,17,19,20,25^, and most codons bias showed higher A/T-ending than G/C-ending^8,17,19,20,25^. Compared to the result of this study, it was confirmed that the 8 chloroplast genomes of *Aglaonema* exhibited the same characteristics as those of reported higher plants^8,17,19,20,25^.

Many studies proved that highly divergent sequences, SSRs and long repeats of chloroplast genome sequences were useful for studies on phylogenetic relationships, species/cultivar identification and molecular markers development^18,26–28^. Research has shown that *Colocasia gigantea*, *Caladium bicolor*, and *Xanthosoma sagittifolium* could be successfully identified with strong support using chloroplast genome sequences, and three DNA barcodes (*atpH-atpI* + *psaC-ndhE*, *atpH-atp*I + *trnS-trnG*, *atpH-atpI* + *psaC-ndhE* + *trnS-trnG*) harboured highly variable regions to distinguish species in the Aroideae subfamily^18^. In the three varieties of *Scutellaria baicalensis*, chloroplast genome can be used as a super-barcode for identification^26^. In *Gleditsia sinensis* and *G. japonica*, the mini-barcode of primers ZJ818F-1038R (*ycf1b*) was proven to precisely discriminate between these two species^27^. In the *Dianthus* species, one valid DNA marker in the *clpP-psbB* region, was used to differentiate *D. caryophyllus*, *D. barbatus*, and two cultivars from *D. superbus*, *D. chinensis*, and one hybrid offspring F1^28^. In this study, seven highly variable regions were detected among 15 chloroplast genomes of *Aglaonema*, including *trnH-GUG-exon1-psbA*, *trnS-GCU-trnG-UCC-exon1*, *trnY-GUA-trnE-UUC*, *psbC-trnS-UGA*, *trnF-GAA-ndhJ*, *ccsA-ndhD*, and *rps15-ycf1-D2* (Fig. 7). Besides the highly variable regions, SSRs and long repeats were also retrieved (Figs. 4 and 5). Among these 8 genomes, SSRs were more frequently located in the LSC regions than in the SSC regions and IR regions (Fig. 5). These findings were in agreement with results from a previous study reported in *A. modestum*^8^. Based on these results, several regions where sequences with high divergence and/or SSR loci were used to develop DNA markers. After PCR and sequencing, we found that 4 DNA markers could be used to differentiate ‘Sapphire’ and ‘White Gem’ from *A. commutatum* ‘San Remo’, ‘Kai Sa’, ‘Pattaya Beauty’, *‘*Silver Queen’, ‘Snow White’, and ‘White Horse Prince’ (Fig. 9, Fig. S4). Therefore, these highly variable regions and SSRs could serve to enrich the molecular marker resources of *Aglaonema* for studying its phylogeny and identification.

In this study, SNPs and indels were also identified among these 8 newly sequenced genomes (Table 3, Tables S6 and S7). It is worth noting that 1 SNP and 1 insertion exist between ‘Sapphire’ and ‘White Gem’ (Table 3). The SNP was located in *psaC*, and the insertion was found in *rps12* (Tables S6 and S7). As we know, ‘White Gem’ was a bud mutation among a population of tissue-cultured ‘Sapphire’ plants (Fig. 1). Therefore, *psaC* and *rps12* genes could be used to differentiate these two cultivars at the molecular level. By comparison, ‘Red Valentine’ versus ‘Hong Jian’ and ‘Red Valentine’ versus ‘Hong Yan’ had no SNPs/indels in a previous study, in which ‘Hong Jian’ and ‘Hong Yan’ were two bud mutations found among tissue-cultured ‘Red Valentine’ plants^8^. These comparisons indicated that the chloroplast genomes may undergo variation between the tissue-cultured mutation of ‘White Gem’ and ‘Sapphire’. Additionally, the other 12 comparison pairs, also contained many SNPs and indels (Table 3, Tables S6 and S7). These SNPs and indels could be used to identify *A. commutatum* ‘San Remo’ and seven green cultivars of *Aglaonema*.

Based on chloroplast genomes, our phylogenetic results strongly supported that the 15 individuals of *Aglaonema* species and cultivars can be classified into two clades, namely, clade I and clade II (Fig. 8, Fig. S3). In a previous study, based on morphological characteristics, the *Aglaonema* genus was classified into two sections, namely, *Aglaonema* and *Chamaecaulon*^1^. By comparison, the clade I and clade II in our phylogenetic trees corresponded to the two sections using morphological classification, namely, *Aglaonema* and *Chamaecaulon*, respectively. Therefore, our phylogenetic results support the morphological classification of the *Aglaonema* genus^1^. In a previous report, the phylogenetic tree based on whole chloroplast genomes strongly supported monophyletic of the *Aglaonema* genus^8^. The reasons may be because this report did not sample plenty of *Aglaonema* species and cultivars. In another study with 54 *Aglaonema* species and cultivars, they were divided into seven clusters by 314 polymorphic amplified fragment length polymorphism (AFLP) markers^2^. This may be because the small cluster in the main cluster was also as a cluster in that study^2^. In fact, from the dendrogram of the 54 *Aglaonema* species and cultivars, there were two main clusters^2^. Regarding the Araceae family, phylogenetic relationships among the 7 subfamilies of Aroideae, Lasioideae, Lemnoideae, Monsteroideae, Orontioideae, Pothoideae, and Zamioculcadoideae, were strongly supported (Fig. 8, Fig. S3). Our phylogenetic trees reconstructed by complete chloroplast genomes for the Araceae family were in agreement with previous studies^8,10,13,14^. In conclusion, there are sufficient complete chloroplast genomes with good reliability to understand the phylogenetic relationships of the *Aglaonema* genus and Araceae family.

In the current study, 34 protein-coding genes with positive selection sites among 77 complete chloroplast genomes of the Araceae family were identified (Tables S9 and S10). Current comparative studies have revealed that our findings revealed more genes under positive selection than the results from the 16 chloroplast genomes of Araceae^8^ and 17 chloroplast genomes of Aroideae^18^, but less genes under positive selection than the results from the 14 chloroplast genomes of Araceae^12^. These differences may be because these three studies used different chloroplast genomes of the Araceae family. These comparisons also reflected the complexity of chloroplast genome evolution in the Araceae family. In the present study, among 34 protein-coding genes with positive selection sites, *ycf2* harboured the highest number of positive amino acids sites (72) (Table S10), suggesting that *ycf2* may play an important role in the adaptive evolution of the Araceae family. Meanwhile, *rpoC2*, *rbcL*, *matK*, *atpA*, *atpB*, *rpoB* and *ndhF* also possessed relatively high positive selection sites (23, 12, 7, 6, 5, 5 and 4, respectively). Recent studies have showed that some of these 34 protein-coding genes under positive selection may be very common in higher plants^13,17,20,23,29–31^. For examples, *rbcL*, *rps8* and *ycf2* have been identified under positive selection in the Monsteroideae subfamily^13^; *ccsA*, *ndhA*, *ndhB*, *rbcL*, *rpoC1*, *rpoC2*, *rps18*, *ycf2* and *ycf4* have been identified under positive selection in the Zingiberoideae subfamily^17^; *ccsA*, *ndhA*, *ndhB*, *psbA*, *psbB*, *psbC*, *rbcL*, *rpoC2*, *rps7*, *atpA*, *atpB*, *rpoA*, *rps3*, *clpP*, *ycf2* and *ycf3* have been identified under positive selection in the *Zingiber* genus^20,29^; *psbA*, *psbB*, *atpA*, *atpB*, *atpF*, *atpI*, *ndhA*, *ndhB*, *ndhC*, *ndhF*, *rps3*, *rps7*, *rps8*, *rps15*, *rpoB*, *rpoC1*, *rpoC2*, *rbcL*, *clpP*, *matK*, *ycf3* and *ycf4* have been identified under positive selection in the Zingiberales order^23^; *rpoC1*, *rpoC2*, *rps15*, *ccsA*, *rbcL*, *ycf2* and *ycf4* have been reported as positive selection in orchid^30^; and *rpoC2*, *atpF*, *atpI*, and *rpl14* have been identified under positive selection in *Allium*^31^. For the one hand, the Araceae species had diverse plant morphologies, such as the perennial herbaceous plants, and epiphytic, climbing shrubs or subshrubs; for example, *A. modestum* was a perennial herb with stem erect, while *Pothos cathcartii* was a climbing subshrub with a length of more than 5 m^3,4^. For the other hand, the Araceae species had different natural habitats; for instance, *A. modestum* lived in dense forests at altitudes of 500–1700 m, whereas *P. cathcartii* was epiphytic on the trunk of dense forests at altitudes of 500–1600 m^3,4^. Therefore, genes of chloroplast genome involved in photosystem, ATP synthase, NADH dehydrogenase and ribosome, may play important roles during the evolution and adaptation of Araceae plants to their natural habitats.

## Conclusion

In this study, 8 complete chloroplast genomes from *A. commutatum* ‘San Remo’*,* ‘Kai Sa’, ‘Pattaya Beauty’, ‘Sapphire’, ‘Silver Queen’, ‘Snow White’, ‘White Gem’ and ‘White Horse Prince’, were sequenced, assembled and reported for the first time. These 8 genomes displayed a typical quadripartite structure and each genome contained 112 different genes, including 79 protein-coding genes, 29 tRNA genes and 4 rRNA genes, with genome lengths of 164,789–166,123 bp. The gene orders, GC contents, codon usage frequency, and IR/SC boundaries showed high degree of conservation. Comparative analyses of 15 complete chloroplast genomes of *Aglaonema* identified 7 highly variable regions, which can be used as potential markers for phylogeny and species identification. Both ML and BI phylogenetic trees based on chloroplast genomes strongly supported that the *Aglaonema* genus was classified into two clades, namely, clade I and clade II. These two clades corresponded to two sections, *Aglaonema* and *Chamaecaulon*, respectively. Based on the highly variable regions and SSRs, 4 DNA markers were developed to differentiate the two clades in *Aglaonema*. Finally, 34 protein-coding genes were under positive selection at levels of amino acids with high posterior probabilities among 77 complete chloroplast genomes of the Araceae family. These results enrich the genomic resources of the *Aglaonema* genus and Araceae family, which are useful for classification of *Aglaonema* and chloroplast genome evolution of Araceae.

## Materials and methods

### Plant materials, chloroplast DNA extraction, and sequencing

Fresh leaves of one species and seven green cultivars of *Aglaonema*, including *A. commutatum* ‘San Remo’*,* ‘Kai Sa’, ‘Pattaya Beauty’, ‘Sapphire’, ‘Silver Queen’, ‘Snow White’, ‘White Gem’, and ‘White Horse Prince’ (Fig. 1), were collected from the resource garden (23°23′ N, 113°26′ E) of the environmental horticulture research institute at the Guangdong Academy of Agricultural Sciences, Guangzhou, China. *A. commutatum* ‘San Remo’ had solid dark green petioles and dark green leaves with medium grey green blotches (Fig. 1A). ‘Kai Sa’ had dark green petioles and blotches and marginal zone green leaves (Fig. 1B). ‘Pattaya Beauty’ had green petioles and leaves with marginal zones dark green and along midribs large grey green blotches (Fig. 1C). ‘Sapphire’ had light red petioles and dark green leaves with midribs and margins red (Fig. 1D). ‘Silver Queen’ had dark green petioles and blotches grey green leaves (Fig. 1E). ‘Snow White’ had white petioles and stripes grey green leaves (Fig. 1F). ‘White Gem’, bud mutation found among a population of tissue-cultured ‘Sapphire’ plants, had white petioles and dark green leaves with midribs and margins white (Fig. 1G). ‘White Horse Prince’ had white petioles and strong yellow-green leaves along midribs and at margins white (Fig. 1H). Each sample was quickly frozen in liquid nitrogen and then stored at − 80 °C until use. Chloroplast genomic DNA was extracted using the modified sucrose gradient centrifugation method^32^. DNA quality and concentration were examined by using 1% (w/v) agarose gel electrophoresis and NanoDrop 2000 microspectrometer (Wilmington, DE, USA). Each qualified DNA was used for construction of a DNA library with fragments of about 350 bp, and then sequenced on an Illumina NovaSeq 6000 platform with 150 bp paired-end reads length (Biozeron, Shanghai, China). The original raw data were checked using FastQC v. 0.11.9 (http://www.bioinformatics.babraham.ac.uk/projects/fastqc/), and then filtered by Trimmomatic v. 0.39^33^ with default settings to delete adaptors and low-quality reads.

## Chloroplast genome assembly and annotation

The remaining high-quality clean reads were de novo assembled using GetOrganelle v. 1.7.6.1^34^ with default settings. Geneious Prime 2022 (Biomatters Ltd., Auckland, New Zealand)^35^ was used for sequence correction with a reference chloroplast genome of *Aglaonema costatum* (MN046881). All the assembled complete chloroplast genomes were annotated using GeSeq^36^ and the online Dual Organellar Genome Annotator (DOGMA)^37^ with default settings, respectively. The transfer RNA (tRNA) and ribosomal RNA (rRNA) sequences were predicted by tRNAscanSE v. 2.0.5^38^ and BLAST v. 2.13.0^39^. The annotated complete chloroplast genomes were first validated using online GB2sequin^40^, and further were verified and formatted using Sequin v. 15.50 from NCBI. The eight annotated complete chloroplast genomes of *Aglaonema* were submitted to GenBank (GenBank accession numbers: *A. commutatum* ‘San Remo’*,* OR068727; ‘Kai Sa’, OR068729; ‘Pattaya Beauty’, OR068726; ‘Sapphire’, OR068730; ‘Snow White’, OR068724; ‘Silver Queen’, OR068725; ‘White Gem’, OR068731; and ‘White Horse Prince’, OR068728) (Table 1). The maps of newly complete chloroplast genomes were drawn using Organellar Genome Draw (OGDRAW) v. 1.3.1^41^.

## Analyses of codon usage, long repeats and SSRs

The codon usage of the 8 chloroplast genomes of *Aglaonema* was detected using MEGA v. 7.0^42^ with default settings. Amino acid frequency was also calculated by the percentage of the codons encoding the same amino acid divided by the total number of codons. Simple sequence repeats (SSRs) were identified using the online MISA-web^43^. SSRs were detected with the thresholds of 10 repeat units for mononucleotides, 5 repeat units for dinucleotides, 4 repeat units for trinucleotides, and 3 repeat units for tetra-, penta- and hexanucleotides. Long repeats including forward, palindrome, reverse and complement repeats, were analyzed using REPuter^44^ with repeat sizes ≧ 30 bp and sequences identity ≧ 90%.

### Comparative genomics analysis in the *Aglaonema* genus

The newly sequenced 8 chloroplast genomes of *Aglaonema* for LSC/IR and SSC/IR boundaries and their adjacent genes were analyzed using IRscope^45^. First, to analyze the differences among the chloroplast genomes of *A. commutatum* ‘San Remo’ and 7 green cultivars of *Aglaonema*, the newly sequenced 8 chloroplast genomes of *Aglaonema* were aligned using MUMmer software^46^ and adjusted manually where necessary by the online Se-Al 2.0 (http://tree.bio.ed.ac.uk/software), using the annotated chloroplast genome of *A. commutatum* ‘San Remo’ as the reference. The single nucleotide polymorphisms (SNPs) and insertion/deletions (indels) among these 8 genomes were recorded separately, as well as their locations in the chloroplast genome. Second, to analyze the differences among the chloroplast genomes of 7 green cultivars of *Aglaonema*, SNPs and indels were also detected, using the annotated chloroplast genome of ‘White Gem’ as the reference.

For the genus *Aglaonema*, the complete chloroplast genome of *A. commutatum* ‘San Remo’ was used as a reference and was compared with the other 14 chloroplast genomes of *Aglaonema*, including the rest of the 7 chloroplast genomes of *Aglaonema* sequenced in this study, and other 7 ones obtained from GenBank (Table S9), using the mVISTA program in the Shuffle-LAGAN mode^47^. The sliding window length was set to 600 bp, and the step size was set to 200 bp. Nucleotide variability (Pi) among these 15 chloroplast genomes of *Aglaonema*, protein-coding genes and intergenic regions were extracted and then calculated using DnaSP v. 6.12.03^48^. Comparisons among these 15 chloroplast genomes of *Aglaonema* were performed using CGView server^49^. GC contents were detected based on GC skew using the equation: GC skew = (G − C)/ (G + C). Additionally, variable and parsimony informative base sites of the LSC, SSC, IRa, IRb, and complete chloroplast genomes were also calculated within the genus *Aglaonema*, respectively.

### Phylogenetic relationships in the *Aglaonema* genus and Araceae family

In order to obtain more detailed and accurate phylogenetic relationships, 77 complete chloroplast genomes both in the *Aglaonema* genus and in other species in the Araceae family were used for construction of the phylogenetic trees (Table S9). *Acorus gramineus* (MN175740), *Acorus tatarinowii* (MN536753), *Acorus americanus* (EU273602), and *Acorus calamus* (AJ879453) were downloaded from the GenBank and used as outgroups. As for the Araceae family, in addition to 15 chloroplast genomes of *Aglaonema* (7 had been reported before and 8 were reported in this study), a total of 62 chloroplast genomes were selected and downloaded from the GenBank, including species from Aroideae, Lasioideae, Lemnoideae, Monsteroideae, Orontioideae, Pothoideae and Zamioculcadoideae in the Araceae family (Table S9). Phylogenetic tree was constructed based on chloroplast genome sequences using Maximum likelihood (ML) and Bayesian inference (BI) methods, respectively. Chloroplast genome sequences were aligned using MAFFT v. 7.458^50^ with default parameters, and manually checked when necessary. The optimal nucleotide substitution model (GTR + G + I) was determined through Akaike Information Criterion (AIC) in jModelTest v. 2.1.10^51^. ML analysis was implemented in PhyML v. 3.0^52^ with 1000 bootstrap (BS) replicates for credibility. BI analysis was performed in MrBayes v. 3.2.6^53^, with two independent Markov Chain Monte Carlo algorithm (MCMC) runs consisting of four Markov chains. Each run was conducted with 200,000 generations, starting from random trees, sampling trees every 100 generations, and discarding the first 10% of samples as burn-in. The final phylogenetic trees were edited and visualized using iTOL v. 6 (http://itol.embl.de/itol.cgi).

### Selective pressure analyses in the Araceae family

To investigate positively selected amino acid sites in the 77 complete chloroplast genomes of Araceae (Table S9), the nonsynonymous (dN) and synonymous (dS) substitution rates of consensus protein-coding genes were calculated by using the CodeML program from PAML^54,55^. Gene selective pressure analysis was based on 61 consensus protein-coding genes sequences after removing all stop codons. The positive selection model of M8 (β & ω > 1) was used to detect positively selected sites based on both the dN and dS ratios (ω) and likelihood ratio tests (LRTs) values^56^. The bayes empirical bayes (BEB) method was used to identify the most likely codons under positive selection, with a posterior probability higher than 0.95 and 0.99 indicating sites under positive selection and strong positive selection, respectively^57^.

### Identification and validation of molecular markers for *Aglaonema* species and cultivars

To identify among the 8 *Aglaonema* cultivars present in this study, we used highly divergent regions and regions containing SSRs of the chloroplast genomes to develop molecular markers. Specific primers were designed using the BatchPrimer 3^58^ with the following parameters: GC content between 40 and 60%, primer length of 18 − 28 bases (average 20 bases), annealing temperature between 54 and 60 °C (average 58 °C) with a maximum discrepancy of 4 °C between the primer pairs and PCR product size of 100 to 500 bp (average 250 bp). PCR reaction system (20 µl) contained 2 µl 10 × PCR buffer, 1 µl dNTP (10 mM each), 0.2 µl Taq DNA polymerase (5U/µl) (TaKaRa, Dalian, China), 0.5 µl forward primer (10 µM), 0.5 µl reverse primer (10 µM), 2.0 µl template DNA (30 ng/µl) and 13.8 µl distilled water. PCR reactions were performed in a T100™ Thermal Cycler (BioRad, USA) as follows: (i) an initial denaturation step at 95 °C for 4 min, (ii) 35 cycles of amplification by denaturation at 95 °C for 30 s, annealing at 54 − 60 °C for 30 s and extension at 72 °C for 1 min and (iii) a final extension at 72 °C for 7 min. PCR products were detected on 1.2% (w/v) agarose gels, and then used for Sanger sequencing (Sangon Biotech, Shanghai, China). After sequencing, multiple nucleotide sequence alignments were carried out using the Muscle alignment module in MEGA v. 7.0^42^ with default parameters.

### Supplementary Information

Supplementary Figures.

Supplementary Tables.

## Data Availability

The data presented in this study are submitted to the NCBI repository (https://www.ncbi.nlm.nih.gov), with accession numbers OR068724 − OR068731. Other chloroplast genomes for phylogenetic analysis can be obtained from NCBI, and their accession numbers are listed in Table S9.

## Abbreviations

bp: Base pairs
BLAST: Basic local alignment search tool
NCBI: National center for biotechnology information
IR: Inverted repeat
LSC: Large single copy
SSC: Small single copy
SSRs: Simple sequence repeats
ML: Maximum likelihood
BI: Bayesian inference
G: Guanine
C: Cytosine
DNA: Deoxyribonucleic acid
RNA: Ribonucleic acid
rRNA: Ribosomal RNA
tRNA: Transfer RNA
Indels: Insertion-deletions
SNPs: Single-nucleotide polymorphisms
PCR: Polymerase chain reaction
RSCU: Relative synonymous codon usage

## References

[1] Nicolson DH (1969). A revision of the genus Aglaonema (Araceae). Smithsonian Contributions to Botany.

[2] Chen J, Devanand PS, Norman DJ, Henny RJ, Chao CCT (2004). Genetic relationships of *Aglaonema* species and cultivars inferred from AFLP markers. Ann. Bot..

[3] Delectis florae reipublicae popularis sinicae agendae academiae sinicae edita. *Flora reipublicae popularis sinicae*. Science press, Beijing, China, **Tomus 13**, 4344 (1979).

[4] Ai TM, Zhang SR, Yang XW, Du LJ, Yan ZY, Dai LK, Zheng YN, Du GH, Li YC (2013). Zhongguo Yaoyong Zhiwuzhi. Zhongguo Yaoyong Zhiwuzhi.

[5] Henny RJ, Chen J, Mellich TA, Brennan MS (2008). Moonlight Bay *Aglaonema*. HortScience.

[6] Henny RJ, Chen J (2009). Key Lime *Aglaonema*. HortScience.

[7] Henny RJ, Chen J (2010). Scenic Bay *Aglaonema*. HortScience.

[8] Li DM, Zhu GF, Yu B, Huang D (2022). Comparative chloroplast genomes and phylogenetic relationships of *Aglaonema modestum* and five variegated cultivars of *Aglaonema*. PLoS One..

[9] Wicke S, Schneeweiss GM, DePamphilis CW, Muller KF, Quandt D (2011). The evolution of the plastid chromosome in land plants: Gene content, gene order, gene function. Plant Mol. Biol..

[10] Henriquez CL, Arias T, Pires JC, Croat TB, Schaal BA (2014). Phylogenomics of the plant family Araceae. Mol. Phylogenet. Evol..

[11] Tian N, Han L, Chen C, Wang Z (2018). The complete chloroplast genome sequence of *Epipremnum aureum* and its comparative analysis among eight Araceae species. PLoS One..

[12] Kim SH (2019). Comparison of whole plastome sequences between thermogenic Skunk Cabbage *Symplocarpus renifolius* and Nonthermogenic *S. nipponicus* (Orontioideae; Araceae) in East Asia. Int. J. Mol. Sci..

[13] Henriquez CL (2020). Evolutionary dynamics of chloroplast genomes in subfamily Aroideae (Araceae). Genomics.

[14] Henriquez CL (2020). Molecular evolution of chloroplast genomes in Monsteroideae (Araceae). Planta.

[15] Abdullah, *et al*. Comparison of chloroplast genomes among species of unisexual and bisexual clades of the monocot family Araceae. *Plants (Basel).* **9**, 737 (2020). 10.3390/plants9060737 PMC7355861 32545339

[16] Abdullah, *et al*. Complete chloroplast genomes of *Anthurium huixtlense* and *Pothos scandens* (Pothoideae, Araceae): unique inverted repeat expansion and contraction affect rate of evolution. *J. Mol. Evol.* **88**, 562574 (2020). 10.1007/s00239-020-09958-w PMC7445159 32642873

[17] Li DM, Li J, Wang DR, Xu YC, Zhu GF (2021). Molecular evolution of chloroplast genomes in subfamily Zingiberoideae (Zingiberaceae). BMC Plant Biol..

[18] Li B (2022). Complete chloroplast genome sequences of three aroideae species (Araceae): Lights into selective pressure, marker development and phylogenetic relationships. BMC Genomics..

[19] Jiang H (2022). Comparative and phylogenetic analyses of six Kenya *Polystachya* (Orchidaceae) species based on the complete chloroplast genome sequences. BMC Plant Biol..

[20] Jiang D (2023). Complete chloroplast genomes provide insights into evolution and phylogeny of *Zingiber* (Zingiberaceae). BMC Genomics..

[21] Rothwell GW, Van Atta MR, Ballard HEJ, Stockey RA (2004). Molecular phylogenetic relationships among Lemnaceae and Araceae using the chloroplast *trnL*-*trnF* intergenic spacer. Mol. Phylogenet. Evol..

[22] Liu S, Wang Z, Wang H, Su Y, Wang T (2020). Patterns and rates of plastid *rps12* gene evolution inferred in a phylogenetic context using plastomic data of ferns. Sci. Rep..

[23] Li DM (2023). Comparative chloroplast genomics of 21 species in Zingiberales with implications for their phylogenetic relationships and molecular dating. Int. J. Mol. Sci..

[24] Mehmetoğlu E, Kaymaz Y, Ateş D, Kahraman A, Tanyolaç MB (2023). The complete chloroplast genome of *Cicer reticulatum* and comparative analysis against relative *Cicer* species. Sci Rep..

[25] Niu Z, Lin Z, Tong Y, Chen X, Deng Y (2023). Complete plastid genome structure of 13 Asian *Justicia* (Acanthaceae) species: comparative genomics and phylogenetic analyses. BMC Plant Biol..

[26] Jiang Y (2023). Identification of three cultivated varieties of *Scutellaria baicalensis* using the complete chloroplast genome as a super-barcode. Sci Rep..

[27] Tan W (2020). The complete chloroplast genome of *Gleditsia sinensis* and *Gleditsia japonica*: genome organization, comparative analysis, and development of taxon specific DNA mini-barcodes. Sci. Rep..

[28] Lin S (2022). Comprehensive comparative analysis and development of molecular markers for *Dianthus* species based on complete chloroplast genome sequences. Int. J. Mol. Sci..

[29] Li DM, Ye YJ, Xu YC, Liu JM, Zhu GF (2020). Complete chloroplast genomes of *Zingiber montanum* and *Zingiber zerumbet*: genome structure, comparative and phylogenetic analyses. PLoS One..

[30] Dong WL, Wang RN, Zhang NY, Fan WB, Fang MF, Li ZH (2018). Molecular evolution of chloroplast genomes of orchid species: Insights into phylogenetic relationship and adaptive evolution. Int. J. Mol. Sci..

[31] Chen J, Xie D, He X, Yang Y, Li X (2022). Comparative analysis of the complete chloroplast genomes in *Allium* Section *Bromatorrhiza* Species (Amaryllidaceae): phylogenetic relationship and adaptive evolution. Genes (Basel)..

[32] Li X (2012). High-throughput pyrosequencing of the complete chloroplast genome of *Magnolia officinalis* and its application in species identification. Acta Pharm. Sin..

[33] Bolger AM, Lohse M, Usadel B (2014). Trimmomatic: A flexible trimmer for Illumina sequence data. Bioinformatics..

[34] Jin JJ (2020). GetOrganelle: A fast and versatile toolkit for accurate de novo assembly of organelle genomes. Genome Biol..

[35] Kearse M (2012). Geneious Basic: An integrated and extendable desktop software platform for the organization and analysis of sequence data. Bioinformatics..

[36] Tillich M (2017). GeSeq—Versatile and accurate annotation of organelle genomes. Nucl. Acids Res..

[37] Wyman SK, Jansen RK, Boore JL (2004). Automatic annotation of organellar genomes with DOGMA. Bioinformatics.

[38] Lowe TM, Chan PP (2016). tRNAscan-SE On-line: Search and contextual analysis of transfer RNA genes. Nucl. Acids Res..

[39] Altschul SF (1997). Gapped BLAST and PSI-BLAST: A new generation of protein database search programs. Nucl. Acids Res..

[40] Lehwark P, Greiner S (2019). GB2sequin—a file converter preparing custom GenBank files for database submission. Genomics.

[41] Greiner S, Lehwark P, Bock R (2019). OrganellarGenomeDRAW (OGDRAW) version 1.3.1: Expanded toolkit for the graphical visualization of organellar genomes. Nucl. Acids Res..

[42] Kumar S, Stecher G, Tamura K (2016). Mega 7: Molecular evolutionary genetics analysis version 7.0 for bigger datasets. Mol. Biol. Evol..

[43] Beier S, Thiel T, Münch T, Scholz U, Mascher M (2017). MISA-web: A web server for microsatellite prediction. Bioinformatics.

[44] Kurtz S (2001). REPuter: The manifold applications of repeat analysis on a genomic scale. Nucl. Acids Res..

[45] Amiryousefi A, Hyvönen J, Poczai P (2018). IRscope: An online program to visualize the junction sites of chloroplast genomes. Bioinformatics.

[46] Marcais G (2018). MUMmer4: A fast and versatile genome alignment system. PLoS Comput. Biol..

[47] Frazer KA, Pachter L, Poliakov A, Rubin EM, Dubchak I (2004). VISTA: Computational tools for comparative genomics. Nucl. Acids Res..

[48] Rozas J (2017). DnaSP 6: DNA sequence polymorphism analysis of large datasets. Mol. Biol. Evol..

[49] Grant JR, Stothard P (2008). The CGView Server: A comparative genomics tool for circular genomes. Nucl. Acids Res..

[50] Rozewicki J, Li S, Amada KM, Standley DM, Katoh K (2019). MAFFT-DASH: Integrated protein sequence and structural alignment. Nucl. Acids Res..

[51] Santorum JM, Darriba D, Taboada GL, Posada D (2014). jmodeltest.org: Selection of nucleotide substitution models on the cloud. Bioinformatics.

[52] Guindon S (2010). New algorithms and methods to estimate maximum-likelihood phylogenies: Assessing the performance of PhyML 3.0. Syst. Biol..

[53] Ronquist F (2012). MrBayes 3.2: Efficient Bayesian phylogenetic inference and model choice across a large model space. Syst. Biol..

[54] Yang Z (2007). PAML 4: Phylogenetic analysis by maximum likelihood. Mol. Biol. Evol..

[55] Gao F, Chen C, Arab DA, Du Z, He Y, Ho SYW (2019). EasyCodeML: A visual tool for analysis of selection using CodeML. Ecol. Evol..

[56] Yang Z (1998). Likelihood ratio tests for detecting positive selection and application to primate lysozyme evolution. Mol. Biol. Evol..

[57] Yang Z, Wong WSW, Nielsen R (2005). Bayes empirical Bayes inference of amino acids sites under positive selection. Mol. Biol. Evol..

[58] You FM (2008). BatchPrimer3: a high throughput web application for PCR and sequencing primer design. BMC Bioinform..

