# Supplementary material for: Comparative chloroplast genomics, phylogenetic relationships and molecular markers development of Aglaonema commutatum and seven green cultivars of Aglaonema
Source: Sci Rep. 2024 May 23;14:11820. doi: 10.1038/s41598-024-62586-y (PMC11116548; doi:10.1038/s41598-024-62586-y)
Supplement: Supplementary file 1 — Supplementary Figures. [file 41598_2024_62586_MOESM1_ESM.docx]

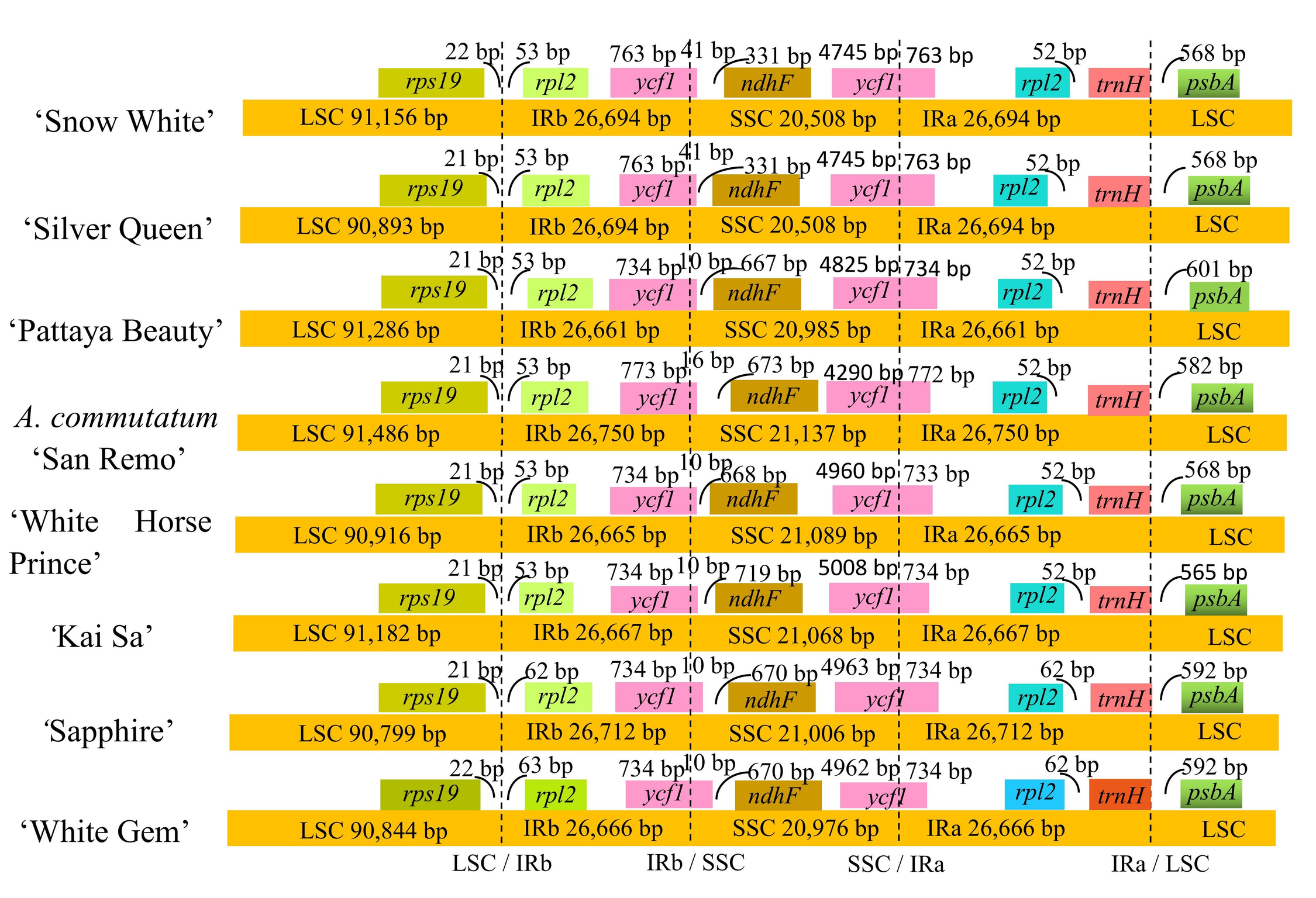


**Figure S1.** Comparison of the IR/SC boundaries among 8 newly sequenced *Aglaonema* chloroplast genomes.

A


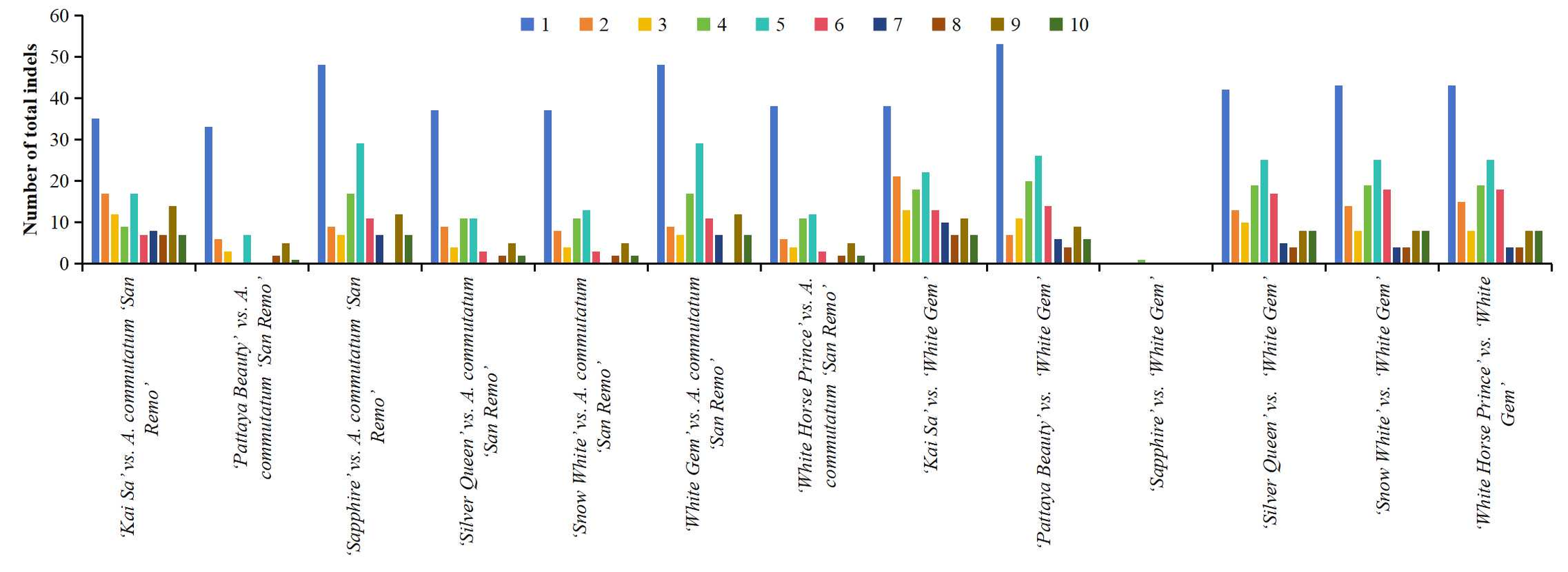


B


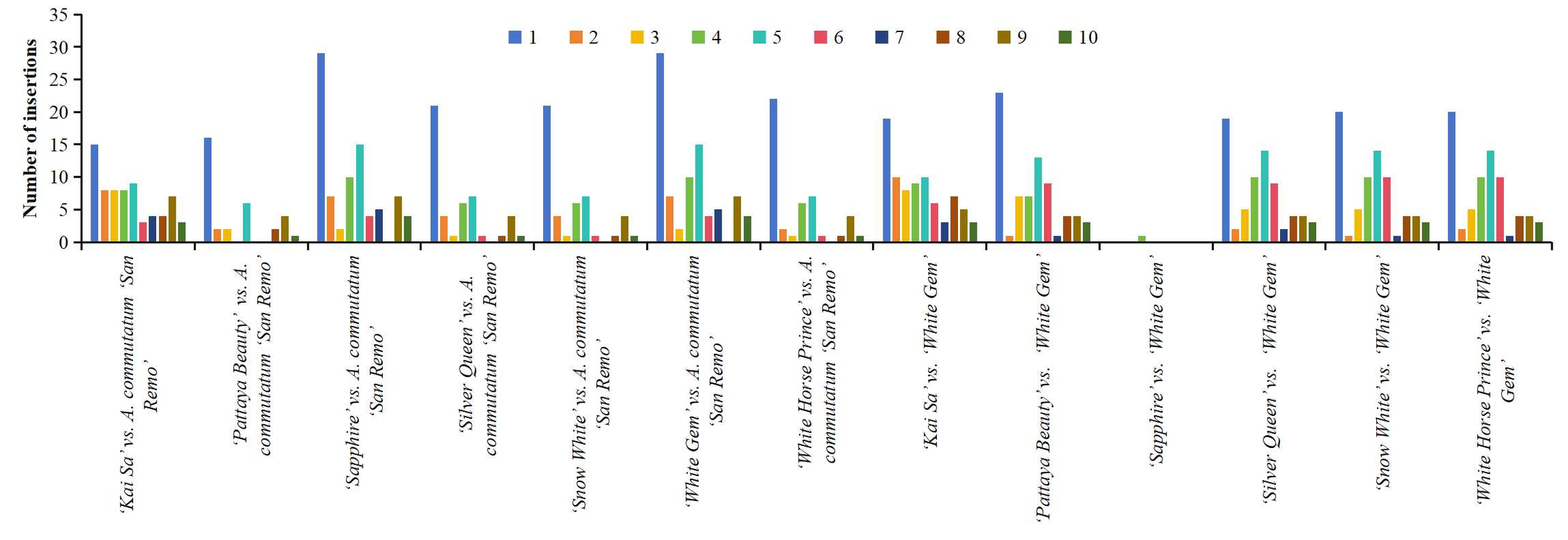


C


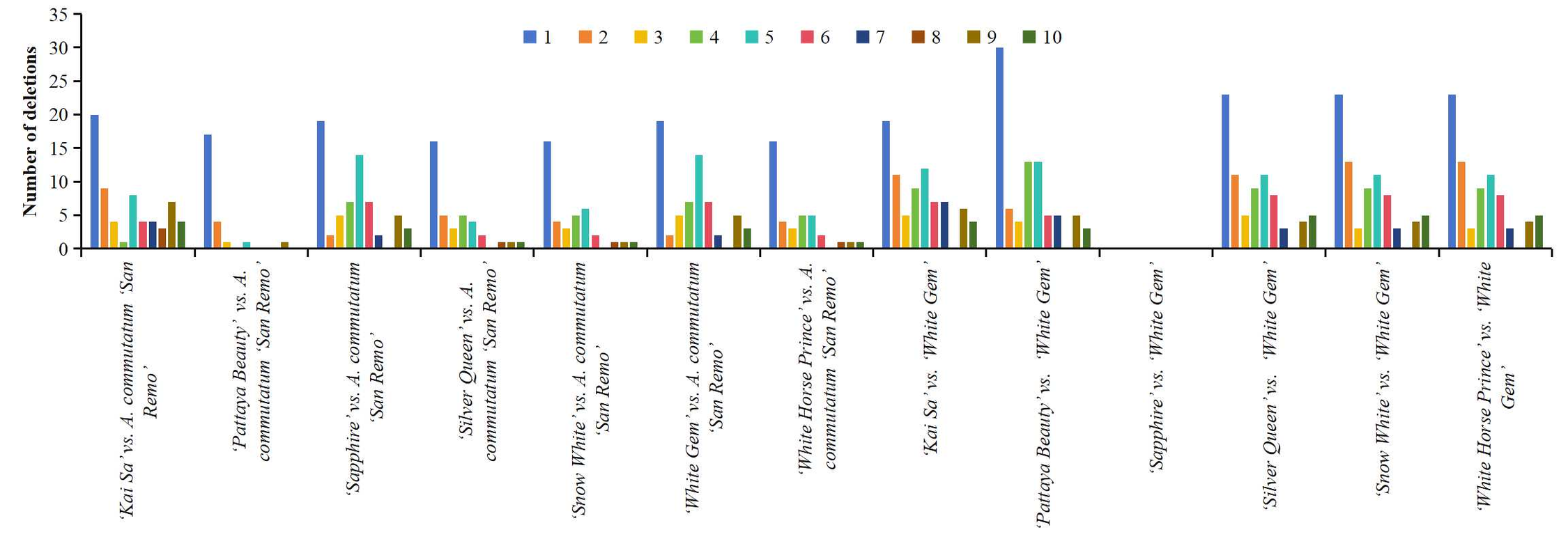


**Figure S2.** Lengths of indels statistics among eight newly sequenced chloroplast genomes of *Aglaonema*. (A) Total indels statistics. (B) Insertion statistics. (C) Deletion statistics.


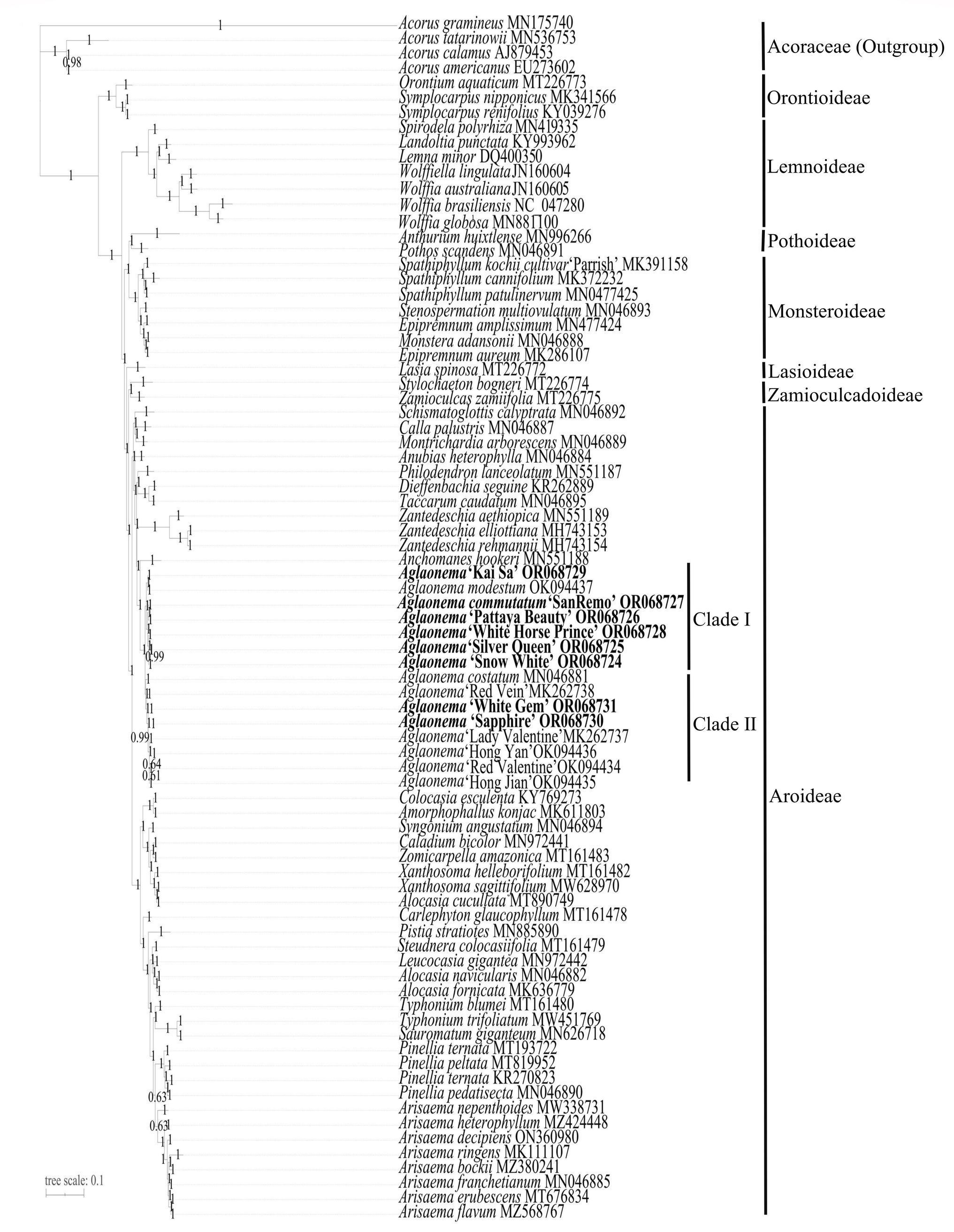


**Figure S3.** Phylogenetic trees of 77 complete chloroplast genomes of the Araceae family using BI method.The 8 newly sequenced *Aglaonema* chloroplast genomes in this study are in bold.

(A)


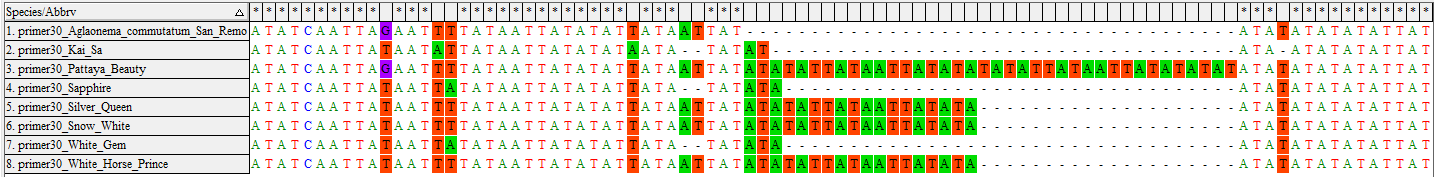


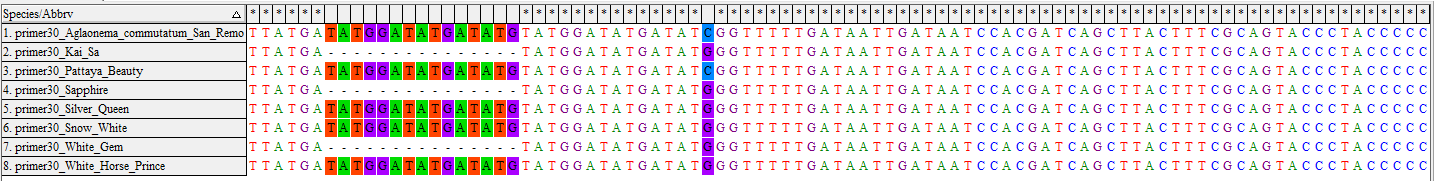


**Figure S4.** Results of multiple nucleotide sequences alignments in different regions after Sanger sequencing. (A)Primer30. (B)Primer83. (C)Primer1. (D)Primer3.

(B)


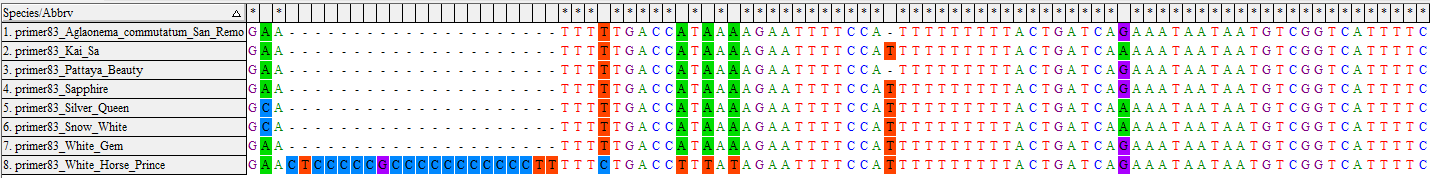


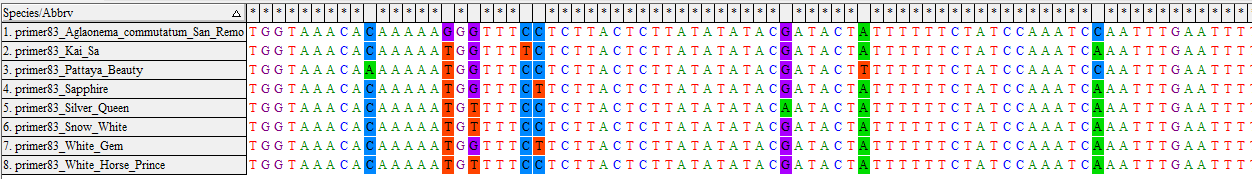


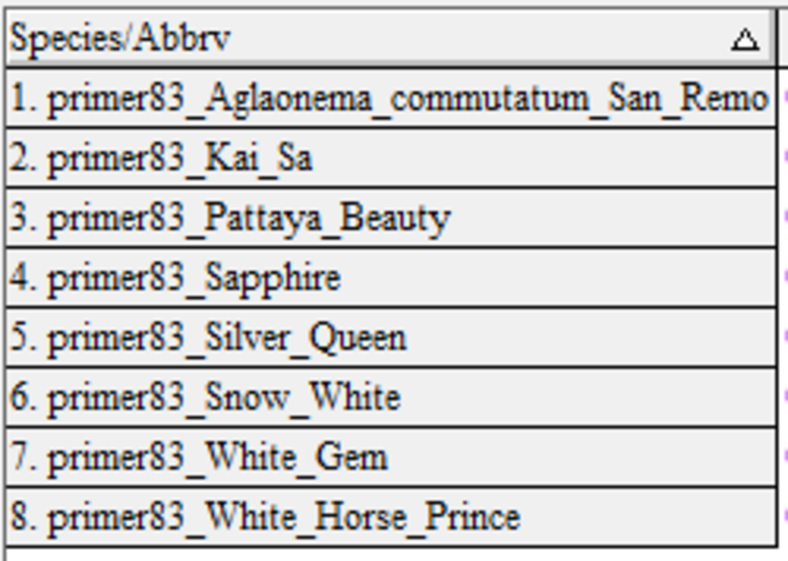

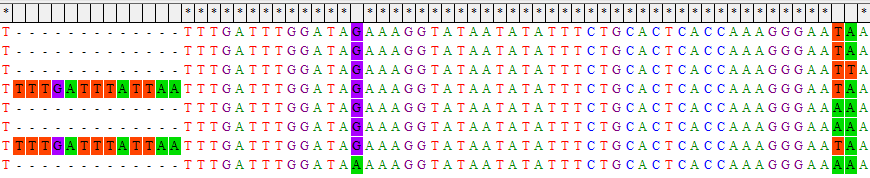


**Figure S4.** Continued.

(C)

**
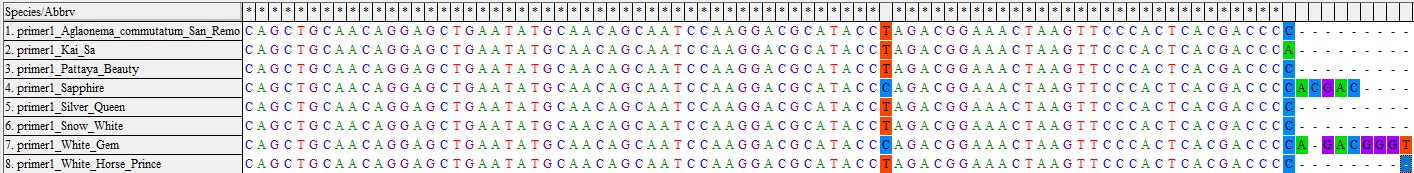
**

(D)


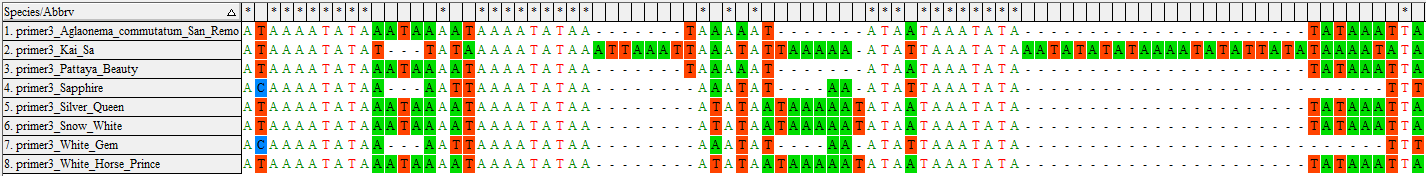


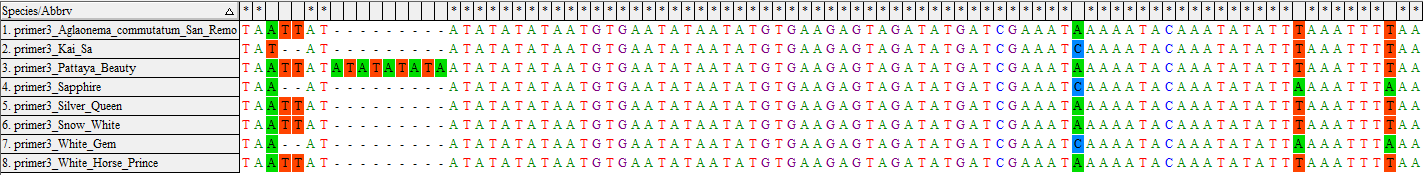


**Figure S4.** Continued.


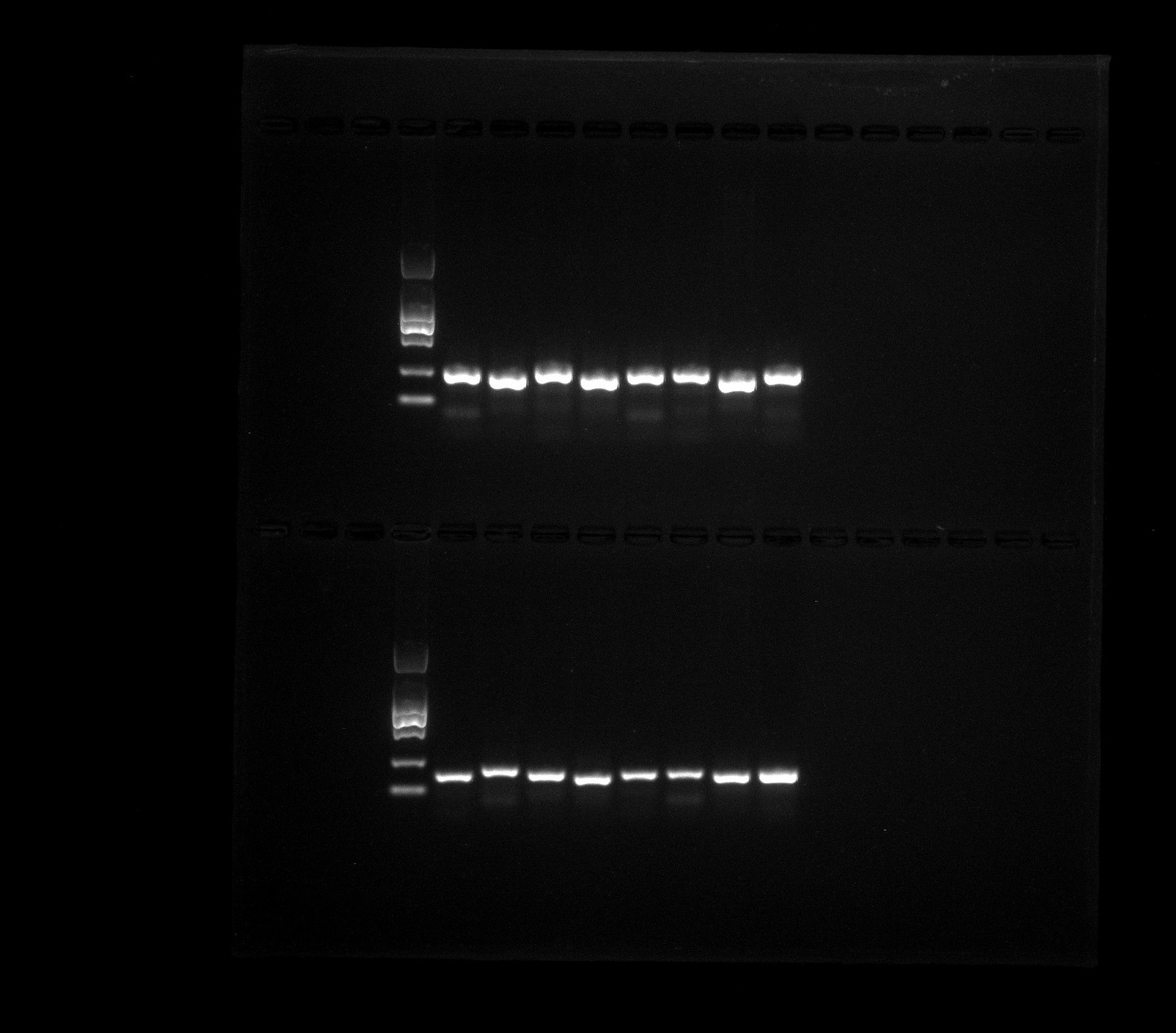


**Supplementary original gel blots of Primer30 and Primer3 in Figure 9.** Primer30 is upper and Primer3 is below. The lanes from left to right, correspond to marker of DL2000, the products amplified from *A. commutatum* ‘San Remo’, ‘Kai Sa’, ‘Pattaya Beauty’, ‘Sapphire’, ‘Silver Queen’, ‘Snow White’, ‘White Gem’, and ‘White Horse Prince’, respectively.


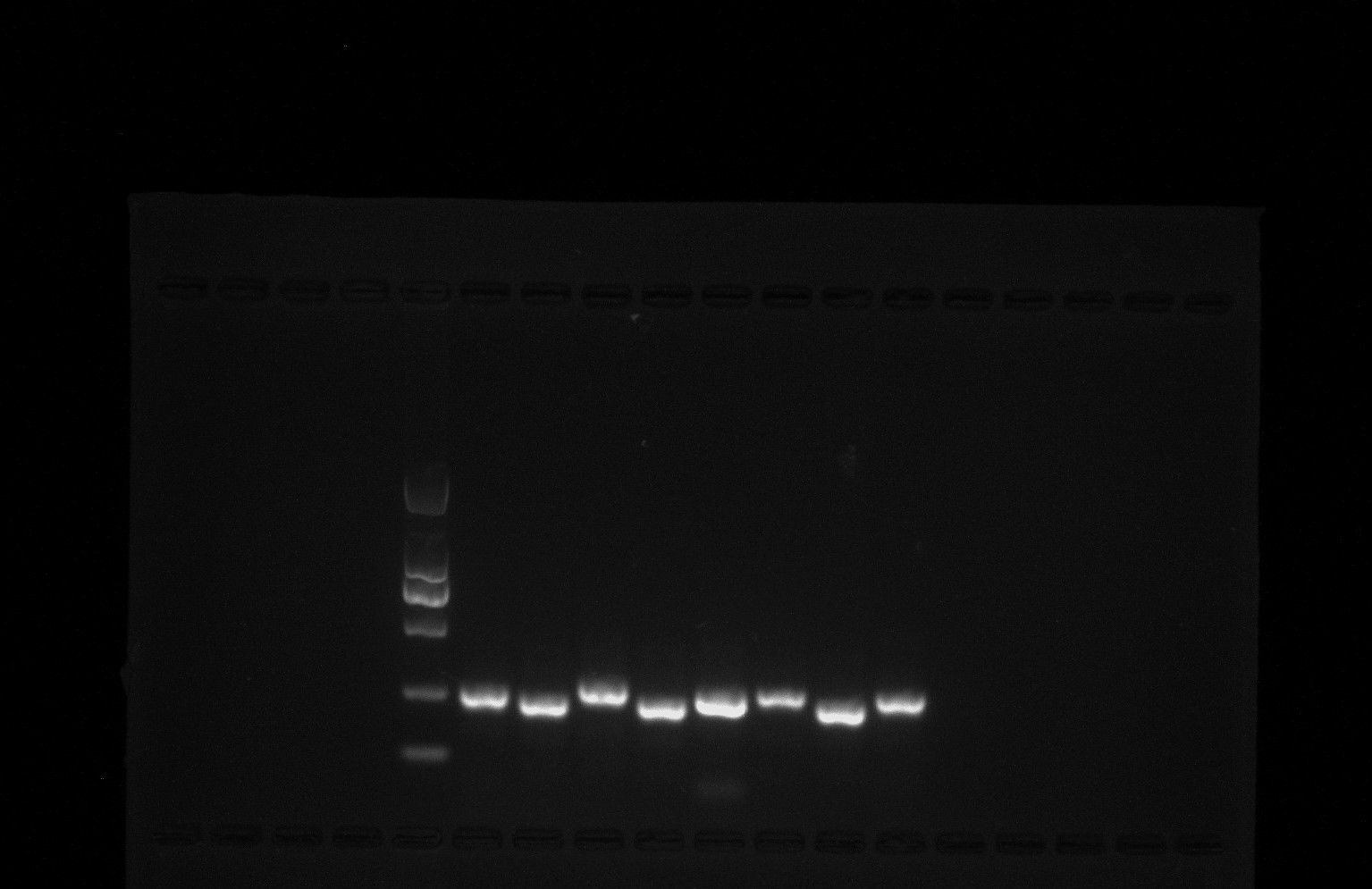


**Supplementary original gel blots of Primer83 in Figure 9.** The lanes from left to right, correspond to marker of DL2000, the products amplified from *A. commutatum* ‘San Remo’, ‘Kai Sa’, ‘Pattaya Beauty’, ‘Sapphire’, ‘Silver Queen’, ‘Snow White’, ‘White Gem’, and ‘White Horse Prince’, respectively.


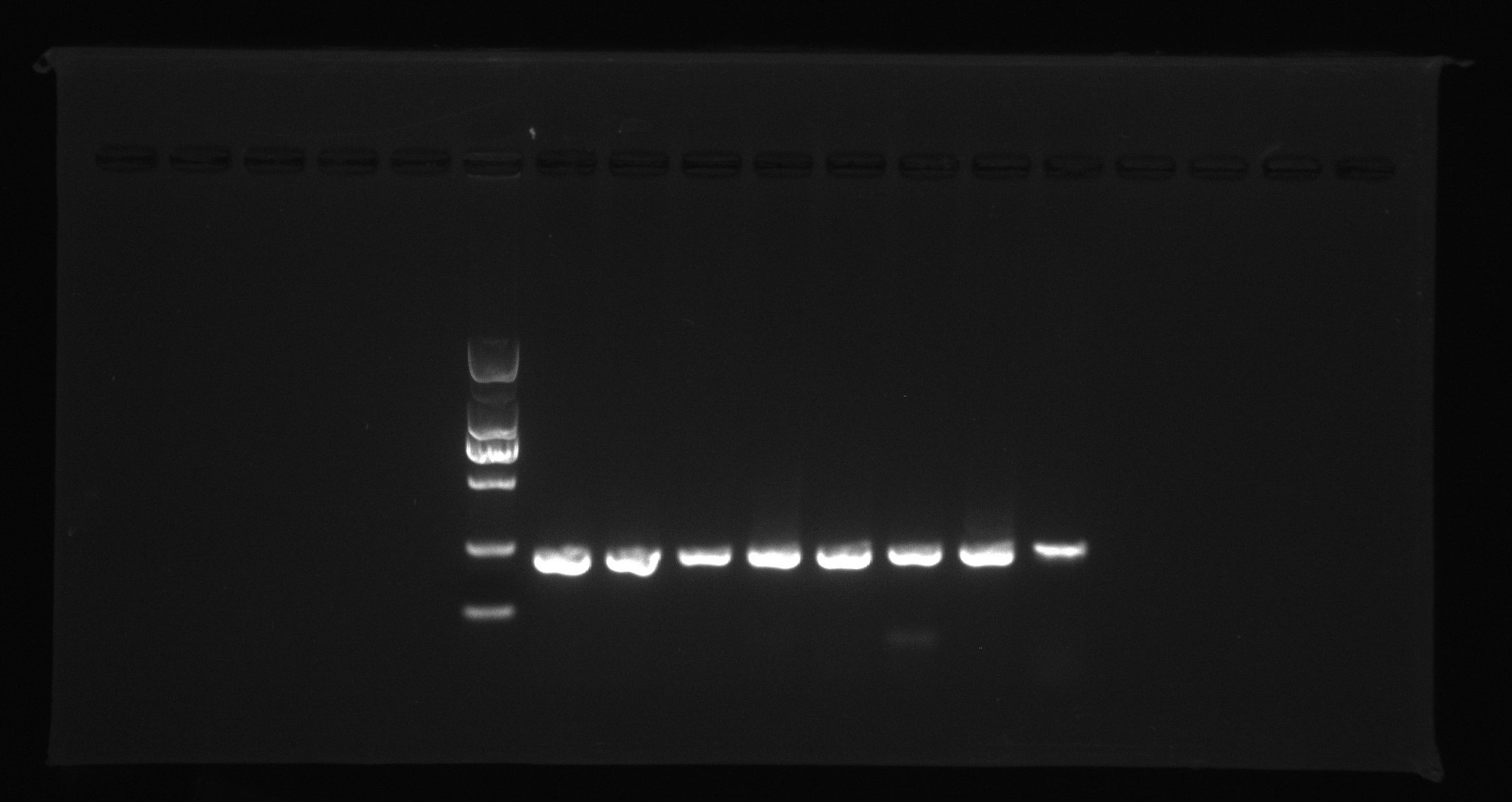


**Supplementary original gel blots of Primer1 in Figure 9.** The lanes from left to right, correspond to marker of DL2000, the products amplified from *A. commutatum* ‘San Remo’, ‘Kai Sa’, ‘Pattaya Beauty’, ‘Sapphire’, ‘Silver Queen’, ‘Snow White’, ‘White Gem’, and ‘White Horse Prince’, respectively.
